# Graph neural network and machine learning analysis of functional neuroimaging for understanding schizophrenia

**DOI:** 10.1186/s12868-023-00841-0

**Published:** 2024-01-02

**Authors:** Gayathri Sunil, Smruthi Gowtham, Anurita Bose, Samhitha Harish, Gowri Srinivasa

**Affiliations:** PES Center for Pattern Recognition, Department of Computer Science and Engineering, PES University, 100 Feet Ring Road, III Stage BSK, Dwaraka Nagar, Bengaluru, Karnataka 560085 India

**Keywords:** Schizophrenia, Graph neural network (GNN), Machine learning, Deep graph convolutional neural network (DGCNN), Biomarkers, Binarization

## Abstract

**Background:**

Graph representational learning can detect topological patterns by leveraging both the network structure as well as nodal features. The basis of our exploration involves the application of graph neural network architectures and machine learning to resting-state functional Magnetic Resonance Imaging (rs-fMRI) data for the purpose of detecting schizophrenia. Our study uses single-site data to avoid the shortcomings in generalizability of neuroimaging data obtained from multiple sites.

**Results:**

The performance of our graph neural network models is on par with that of our machine learning models, each of which is trained using 69 graph-theoretical measures computed from functional correlations between various regions of interest (ROI) in a brain graph. Our deep graph convolutional neural network (DGCNN) demonstrates a promising average accuracy score of 0.82 and a sensitivity score of 0.84.

**Conclusions:**

This study provides insights into the role of advanced graph theoretical methods and machine learning on fMRI data to detect schizophrenia by harnessing changes in brain functional connectivity. The results of this study demonstrate the capabilities of using both traditional ML techniques as well as graph neural network-based methods to detect schizophrenia using features extracted from fMRI data. The study also proposes two methods to obtain potential biomarkers for the disease, many of which are corroborated by research in this area and can further help in the understanding of schizophrenia as a mental disorder.

**Supplementary Information:**

The online version contains supplementary material available at 10.1186/s12868-023-00841-0.

## Background

Schizophrenia is a chronic neurodevelopmental disorder displaying aberrations in the functional connectivity of the brain that affects around 2% of the world’s population [1]. It is characterized by symptoms like hallucinations, disordered thinking, and disorganized speech. Due to an incomplete understanding of the neuropathology of the disorder and the existence of subtle variations from patient to patient, obtaining a reliable diagnosis has become challenging and can lead to misdiagnosis [2, 3]. Fortunately, it has been found [4] that detection of schizophrenia in the prepsychotic prodromal stage followed by cognitive behavioural therapy in the same stage favourably influences the course of the illness. Our study capitalizes on the advantageous aspect that early schizophrenia detection offers, as it holds the potential to enhance treatment outcomes.

A popular modality used in researching cognitive brain functions and psychiatric disorders like schizophrenia is resting state fMRI (rs-fMRI) [5]. The high-level functional organization of the brain can be captured by modeling the brain as an intricate network of nodes and edges based on the rs-fMRI scans (which measure the blood-oxygen-level-dependent (BOLD) signals in the brain), and the topological relationships of the brain can be studied using graph theory [6–8]. Due to the rapid emergence of machine learning (ML) in the healthcare domain, a combination of ML and graph theory has shown promising results for this cause [9, 10]. Several studies have utilized ML models ranging from mathematically simple models such as Logistic Regression to more complicated ones such as ensemble learning techniques [9, 11, 12].

Since brain graphs in their present form constitute a non-Euclidean dataset, their structural, as well as nodal characteristics and their underlying patterns in the context of a neurological framework, may not be completely encapsulated via conventional machine learning models. Recently, graph convolutional networks (GCNs) have been used in an attempt to more precisely detect the intricate and abstract interactions of brain networks [13, 14]. Other neurological disorders, like bipolar disorder, depression, and Alzheimer’s disease, have also been studied with the help of graph neural networks (GNNs) [15, 16]. Several studies on fMRI analysis using GNNs have also been undertaken as specified in [17–19] Another area where work has been done to improve the understanding of schizophrenia is that of biomarker detection using feature selection, multivariate analysis, deep learning, etc. [20, 21].

Building upon this evaluation of prior research, our study encompasses the following key undertakings:

- Graph theory, machine learning, and graph representational models are utilized to comprehend and identify schizophrenia.
- A deep graph convolutional neural network (DGCNN) architecture is introduced for the classification of schizophrenic and control subjects.
- Due to the sensitive nature of its application, the interpretability of the models is enhanced using GNNExplainer, univariate feature analysis, and Shapley Additive explanation (SHAP) values.
- Two innovative feature selection methods are presented- RLF feature selection and SpeCo- for identifying biomarkers in the form of Regions of Interest (ROIs) of the brain, which aids in differentiating the brain characteristics of schizophrenic subjects from those of control subjects.

**Fig. 1:**
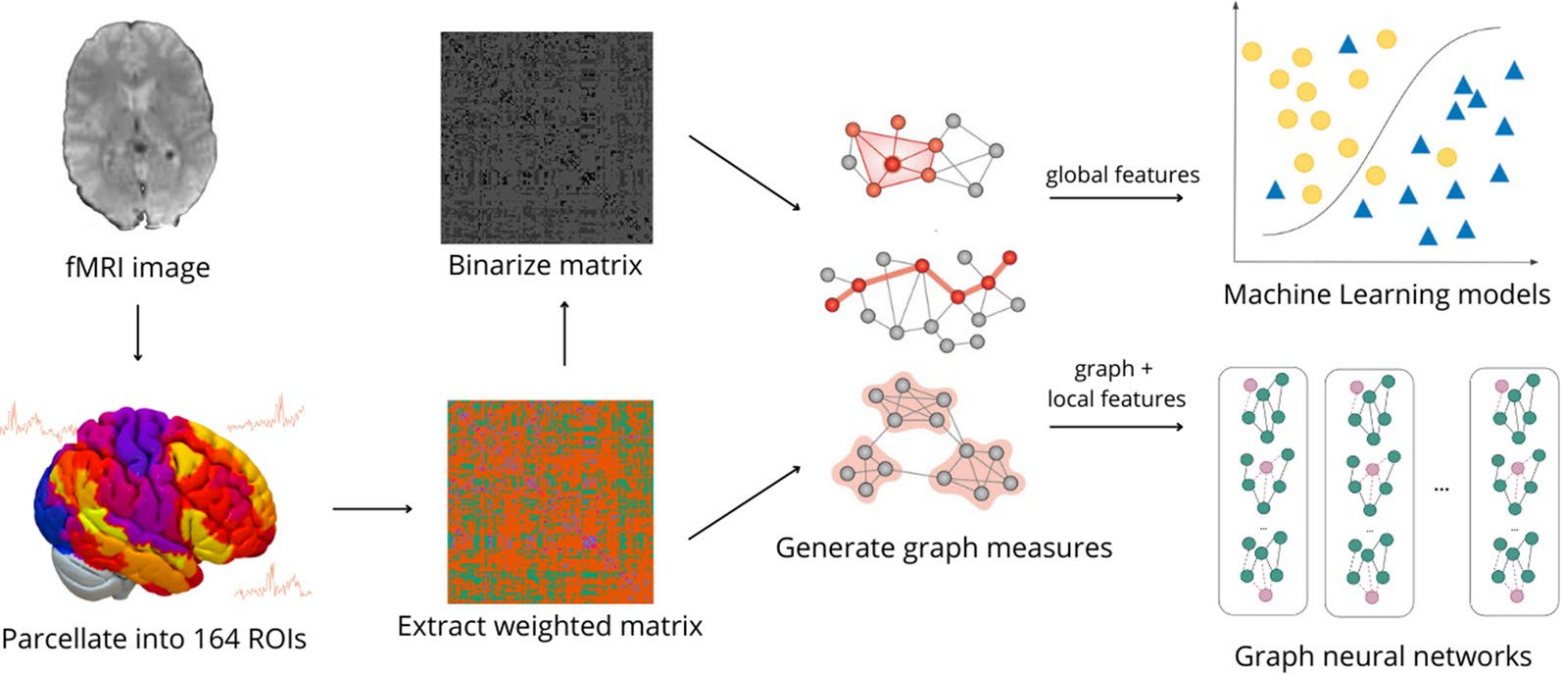
Workflow of the current study

Figure 1 shows the high-level sequence of steps that has been carried out in this study.

## Materials and methods

### Data

The fMRI data has been acquired from the publicly available UCLA Consortium for Neuropsychiatric Phenomics LA5c study which contains 50 schizophrenic subjects and 122 control subjects [22]. The data used has both the structural and the functional MRI scans for all the subjects. The data is imbalanced (with more control subjects) and this has been addressed via data augmentation as described in “Data augmentation” section. The dataset can be downloaded from OpenNeuro repository. More information about the dataset acquisition can be found here and in Additional file 1: Sect. S2.

### Preprocessing

The fMRI data has $60\times 60\times 30= 108,000$ voxels captured over a specified duration of time (TA). Preprocessing is vital to remove noise, account for head motion of the patient, and correct other idiosyncrasies present. For this purpose, we use a preprocessing pipeline with the following steps: functional realignment, slice timing correct, outlier detection, segmentation, normalization, functional smoothing, and temporal bandpass filtering.

- Functional realignment: The first step in the preprocessing pipeline is functional realignment and unwarping in which all scans are co-registered and resampled to a reference image (first scan) using b-spline interpolation. This corrects any distortions arising from the head motion of the patient.
- Slice timing correction: The second step is slice timing correction in which any timing-related misalignment between different slices of the functional data due to delays is corrected and adjusted.
- Outlier detection: The third step is outlier detection which identifies potential outliers based on the BOLD (Blood Oxygen Level Dependent) signal and head motion of the patient by checking frame-wise displacement and looking for any spikes in the values.
- Segmentation and Normalization: The fourth step is segmentation and normalization where each of the anatomical images is segmented into 3 parts—cerebrospinal fluid tissue, grey matter, and white matter. The functional and anatomical data are resampled to voxels of size $2\,{\hbox {mm}}^3$ and $1\,{\hbox {mm}}^3$ respectively.
- Functional Smoothing: The fifth step removes noise from each voxel’s time series signal using a convolution operation and the filter used is a Gaussian kernel of size 8 mm. This improves the peak signal-to-noise ratio (PSNR).
- Temporal bandpass filtering: The last step is denoising where we pass each signal through a temporal bandpass filter of size (0.01, 0.08).

**Fig. 2:**
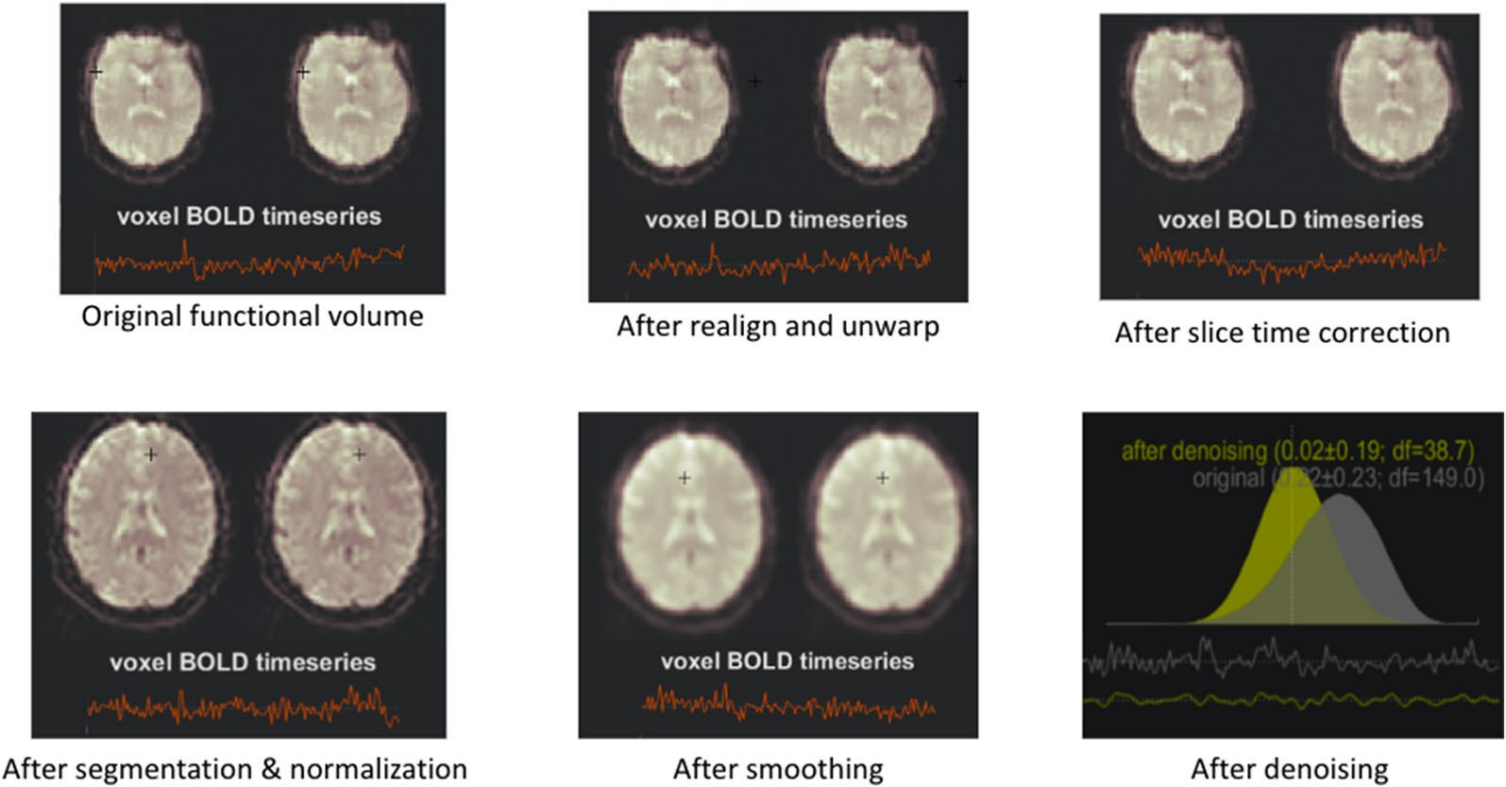
Preprocessing pipeline

At this stage, we possess preprocessed time series signals per voxel for each subject. Because ROI-focused techniques have demonstrated greater effectiveness compared to voxel-oriented methods [23], we have taken an additional measure of combining the time series data from all voxels within individual regions and calculating the average to derive a singular time series for each region. This process also assists in addressing the challenge of high dimensionality, given that each subject comprises 108,000 voxels. For this purpose, we parcellate the brain into 164 regions in accordance with AAL3 Atlas [24] and Harvard-Oxford Cortical Atlas [23]. The complete list of ROIs and their corresponding coordinates can be found in Additional file 1: Sect. S1. Figure 2 depicts the intermediate results for one subject through each stage of the preprocessing pipeline.

### Creation of brain network

In order to formulate graphical analysis of different connections among regions of interest (ROIs) of a brain, and to quantify the functional connectivity between these ROIs, fMRI in itself does not suffice. It needs to be converted to a different representation. The most common technique used for this purpose is to correlate regions of the brain using a measure called Pearson correlation coefficient(PCC) (1). First, PCC is computed for each pair of ROIs. Then, the inverse tanh function is applied to these intermediate values to get the Fisher-transformed bi-variate correlation coefficients as defined in (2). Fisher-transformed correlation coefficient values normalise the Pearson correlation coefficient values. All negative correlation values are set to 0 [25, 26] due to the indefinite biological interpretation of negative functional connectivity in the brain.

1 $$\begin{aligned} r(i,j)&= \frac{\int R_{i}(t)R_{j}(t)dt}{(\int R^{2}_{i}(t)dt \int R^{2}_{j}(t)dt)^{1/2}} \end{aligned}$$

2 $$\begin{aligned} Z(i,j)&= \tanh ^{-1}(r(i,j)) \end{aligned}$$

where

- r is a matrix of Pearson Correlation Coefficients
- R is the BOLD time series within each ROI and
- Z is the matrix of Fisher-transformed correlation coefficients.

**Fig. 3:**
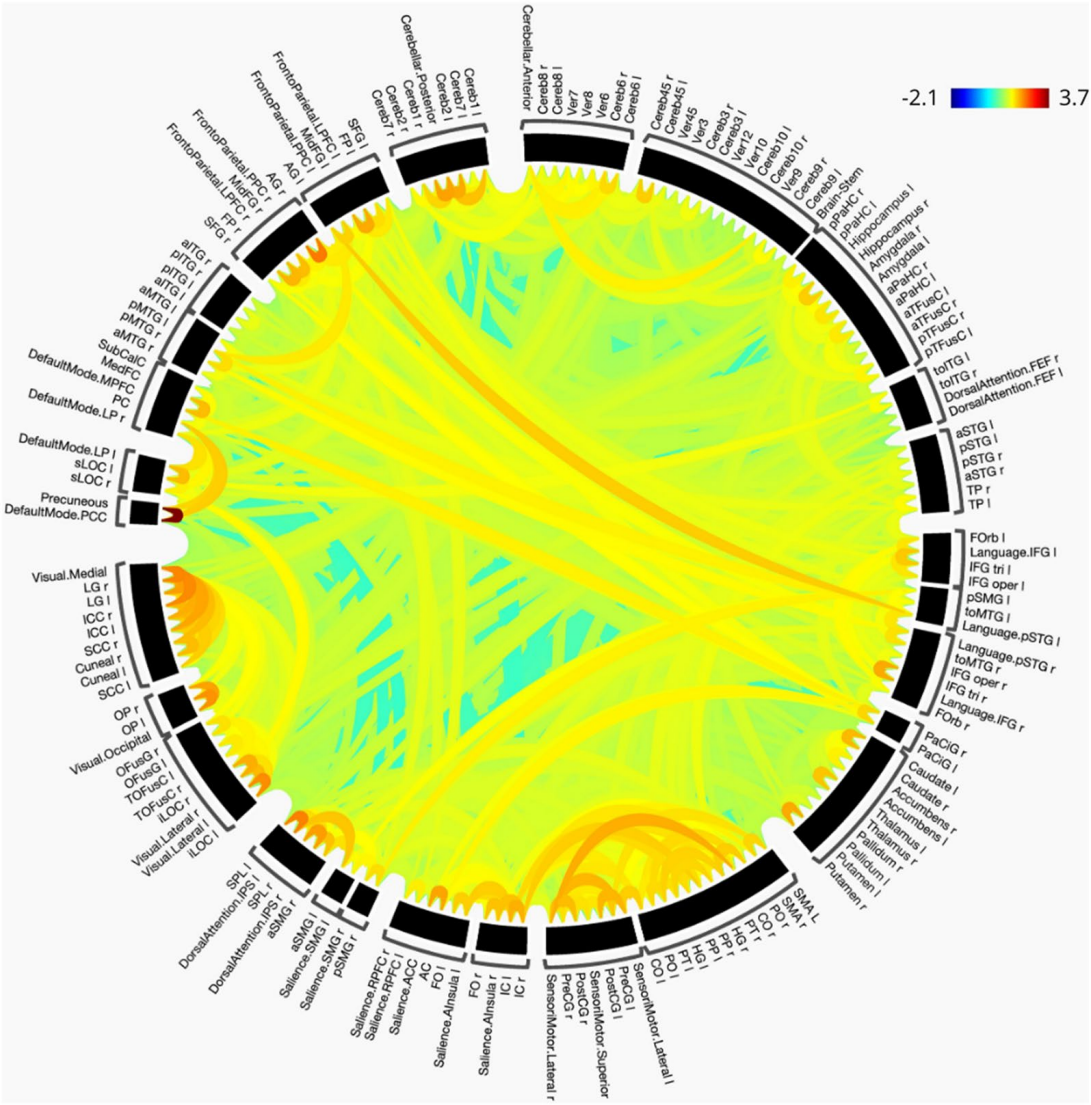
Functional correlation between different ROIs

The adjacency matrix (r) thus formed is a $164 \times 164$ weighted matrix which is a mathematical representation of the weighted graph of the brain, also referred to as a “brain graph”. From a geometric perspective, this brain graph contains 164 nodes where each distinct node corresponds to one of the 164 distinct ROIs in the brain, and, the edge between each pair of ROIs has a weight equivalent to the functional correlation between the two ROIs. This results in 164x164 different edges in a brain graph, and this is what is represented by the adjacency matrix (r). Figure 3 shows a visual representation of the correlation between brain regions or ROIs.

#### Binarization of brain network

**Fig. 4:**
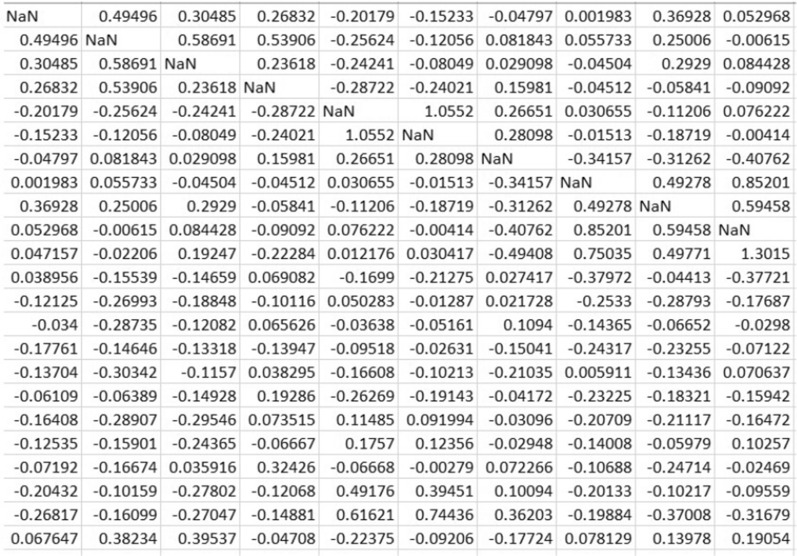
Weighted matrix

**Fig. 5:**
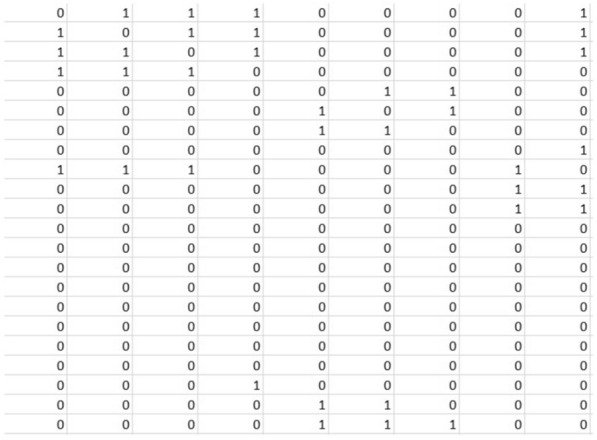
Binary matrix

Binarizing a weighted brain graph converts the weighted connections or edges between nodes in the brain graph into binary values by applying a threshold to the weights. This process transforms the continuous or weighted network into a binary representation, where edges are either present (assigned a value of 1) or absent (assigned a value of 0) based on the chosen threshold for the weights. We have taken this additional step of converting each of the generated weighted graphs into corresponding unweighted graphs using 9 fixed thresholds: 0.00, 0.05, 0.10, 0.15, 0.20, 0.25, 0.30, 0.35, 0.40. Both the weighted and unweighted graphs maintain an undirected nature. As detailed in “Preliminary analysis” section, we have evaluated the classification performance for each of these thresholds to identify the optimal fixed threshold for binarizing the matrices. The graphs that have been transformed using this determined threshold will be utilized for the remainder of the study. Figure 4 shows a weighted adjacency matrix which, when binarized using a threshold of 0.20, creates a binary matrix shown in Fig. 5.

### Feature generation

Seed-based connectivity is one of the methods that has been employed by us in order to understand and analyse functional connectivity of the brain. These measures are computed for each ROI in the brain graph. Hence, to capture the intricacies of the brain graphs and to generate adequate features for the machine learning and graph neural network models, we have calculated 69 graph measures belonging to 2 broad categories: nodal and global. Nodal or local features give information about node connectivity and the importance of certain nodes in the brain. 26 nodal features have been generated for each of the 164 ROIs per subject, which gives $26\times 164= 4264$ features per subject. Global features give us insights into the graph as a whole and generate one value per subject. Within each category, features can be further segregated into binary and weighted, based on the matrix used for their generation. Table 1 provides a summary of the features generated. The definitions of all the 69 graph features can be found in Additional file 1: Sect. S3.

**Table 1:** Summary of generated graph properties

No.	Category	# Features	Examples
1	Global binary	36	Degree assortativity, # cliques, transitivity
2	Global weighted	7	Dijkstra path length, Wiener index, conductance
3	Local binary	21	Centrality (degree, betweenness, closeness, load)
4	Local weighted	5	Closeness vitality, effective size, weighted degree

### Preliminary analysis

**Fig. 6:**
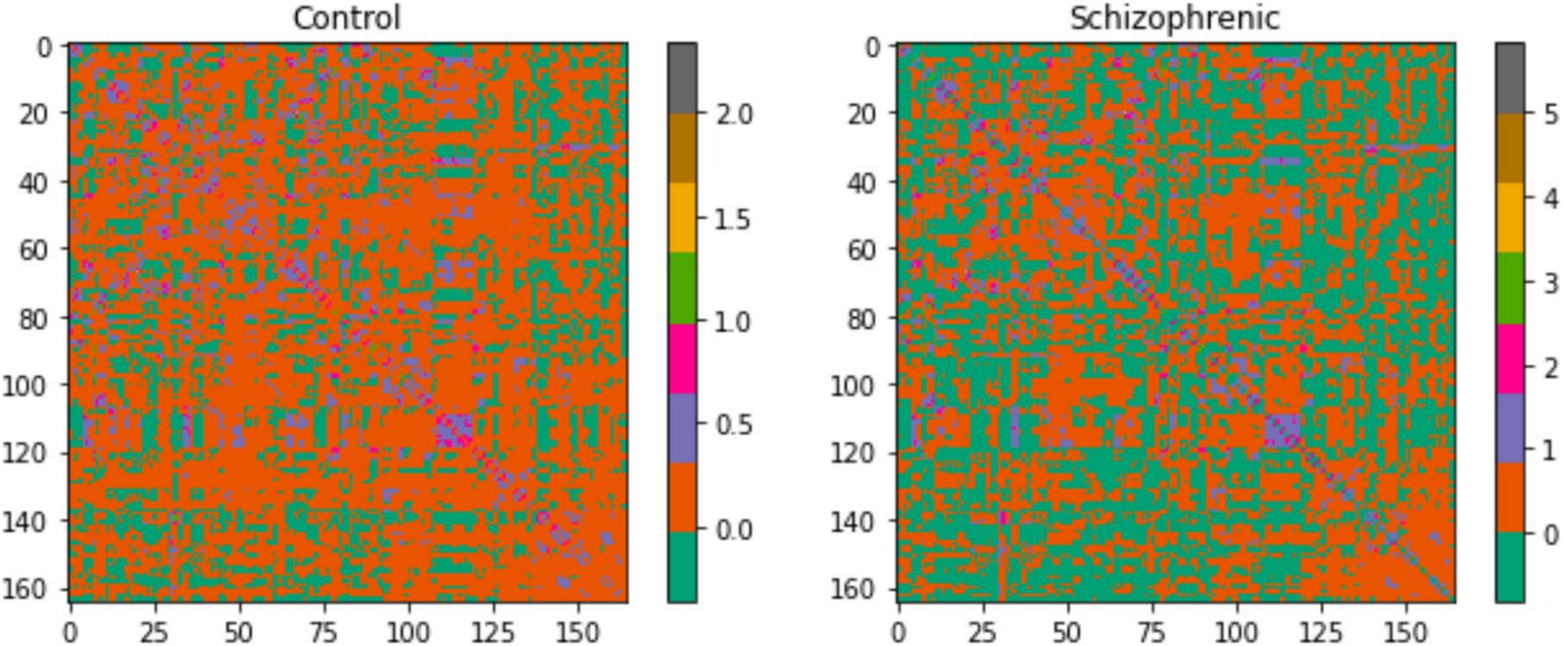
Heatmaps of weighted matrices

We have initiated the initial examination of the data through the execution of exploratory data analysis (EDA) and visualization of the brain graphs. As an illustration, Fig. 6 depicts a qualitative heatmap showcasing the average weighted matrices of both control and schizophrenic subjects. This visualization method assists in discerning the subtle variations in weights by employing a distinct color scheme for contrast.

Further, feature-based analysis has been carried out, yielding interesting results. We have conducted this analysis at two levels: global and nodal.

- At the global level, the average maximal cliques for schizophrenic subjects are found to be 2.5–3 times higher than in control subjects. The average clustering coefficient is also found to be lower in schizophrenic subjects than in control subjects and the average shortest path length is found to be higher in schizophrenic subjects which is consistent with [27].
- From a nodal perspective, a comparison has been made by first averaging each nodal feature for every ROI of all the subjects belonging to each of the two categories. This leads to $26\times 164$ i.e., 4, 264 features for schizophrenic and control subjects respectively. Then, a comparison has been drawn between the average number of cliques that each ROI belongs to. 46 ROIs are contained in more than 220% cliques in schizophrenic subjects as compared to control subjects. 90 ROIs are contained in more than 170% cliques in schizophrenic subjects as compared to control subjects.

### Comparison of binarizing thresholds

**Fig. 7:**
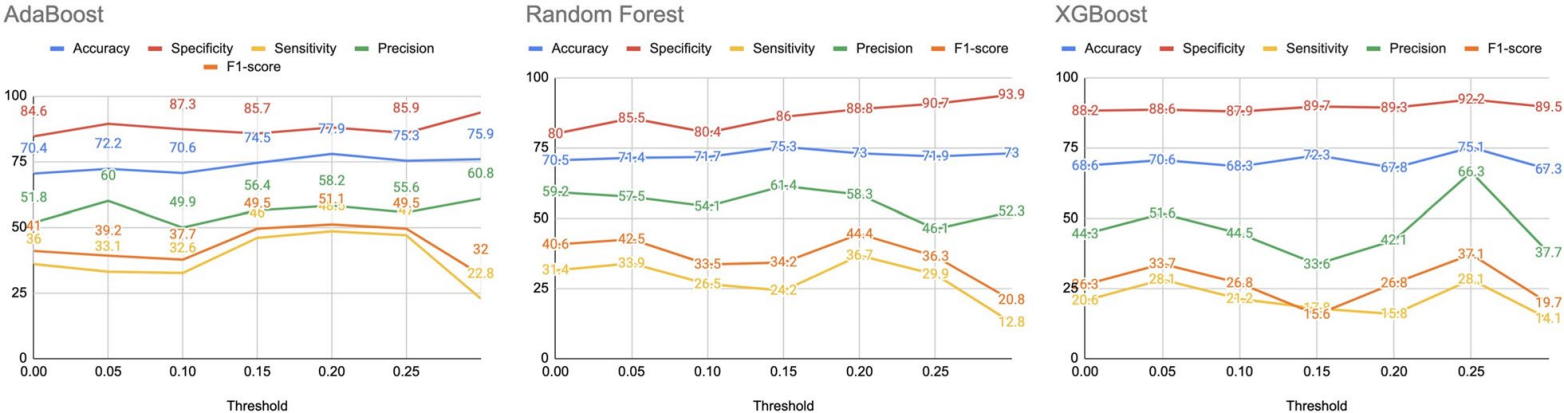
Variation of accuracy, specificity,sensitivity,precision,f1-score for AdaBoost, Random Forest and XGBoost

The brain network obtained for each subject is a weighted adjacency matrix. We binarize each weighted brain graph by transforming the original graph which has weighted connections (edges) between nodes, into a binary graph where edges are either present or absent (1 or 0) based on a threshold. To determine the binarizing threshold that best captures the subtleties of the brain network, we compare the efficacy of various thresholds ranging from 0.00 to 0.40 in increments of 0.05. 36 global binary measures have been generated per threshold value for all 172 subjects. However, the number of disconnected graphs (with isolates) starts increasing beyond 0.35 (at the threshold of 0.35, 42 subjects have disconnected graphs and at the threshold of 0.40, 115 subjects have disconnected graphs), causing a hindrance to the calculation of certain graph features. Therefore, the comparison is limited to the threshold value of 0.30. We employ 3 machine learning models: Random Forest, XGBoost, and AdaBoost to compare the performance of these thresholds. The 3 machine learning models are trained and tested on the global binary adjacency matrices of the schizophrenic and control patients. 10-fold Grid Search Cross Validation is used for all models and evaluated using 5 metrics, namely, accuracy, specificity, sensitivity, precision and f1-score. The complete list of results can be found in Additional file 1: Sect. S4. While no single threshold is statistically better than other thresholds, 0.2 yields the most promising result in terms of the average metrics as can be visualized in Fig. 7. As a result, we have used the features of graphs binarized at 0.2 for the remainder of the study.

### Data augmentation

Class imbalance in the data can lead to biased learning in the models [28, 29]. Moreover, neural networks have been known to work better with larger datasets. To address this issue, a data augmentation technique is employed where 5 pairs of ROIs are randomly sampled from each subject and a uniform noise in the range of (0.0, 0.3) is added. This is equivalent to perturbing 10 values out of $164\times 164$ possible values i.e., 0.037% of the values. This small number has been chosen with the intent of preserving most properties of a brain graph while also generating more data to increase the robustness of the model. The labels for these are generated using 2 ML models: Random Forest and XGBoost which are trained on the original data. A collective decision made by the 2 ML models is considered for the final label, and the synthetic matrices having a tie in the votes are dropped.

### Machine learning

We have used 5 ML models for classification: AdaBoost, Decision Tree, K Nearest Neighbours (KNN), Support Vector Machine (SVM), and Logistic Regression. Random Forest and XGBoost have not been employed to prevent any possible bias from re-using the same models that have been used to predict the labels for the augmented data. The hyperparameters defined for each ML model can be found in Additional file 1: Sect. S5.

First, the feature dataset containing global graph measures is passed through a standardization and normalization pipeline for preprocessing. GridSearchCV is used to get optimal hyperparameters and to test the model on different train-test splits. The average metrics with significance have been reported in Table 2.

### Graph neural networks (GNN)

GNNs can directly use non-Euclidean data as input and leverage network-level information via nodal features and neighborhood relationships. For each GNN model, we have used the binary graphs along with binary and weighted nodal properties as input. 2 different GNN architectures have been used. Early stopping mechanisms have been used in training, along with an Adam optimizer. Both variants of the GNN models perform supervised graph classification.

#### Graph convolutional network

**Fig. 8:**
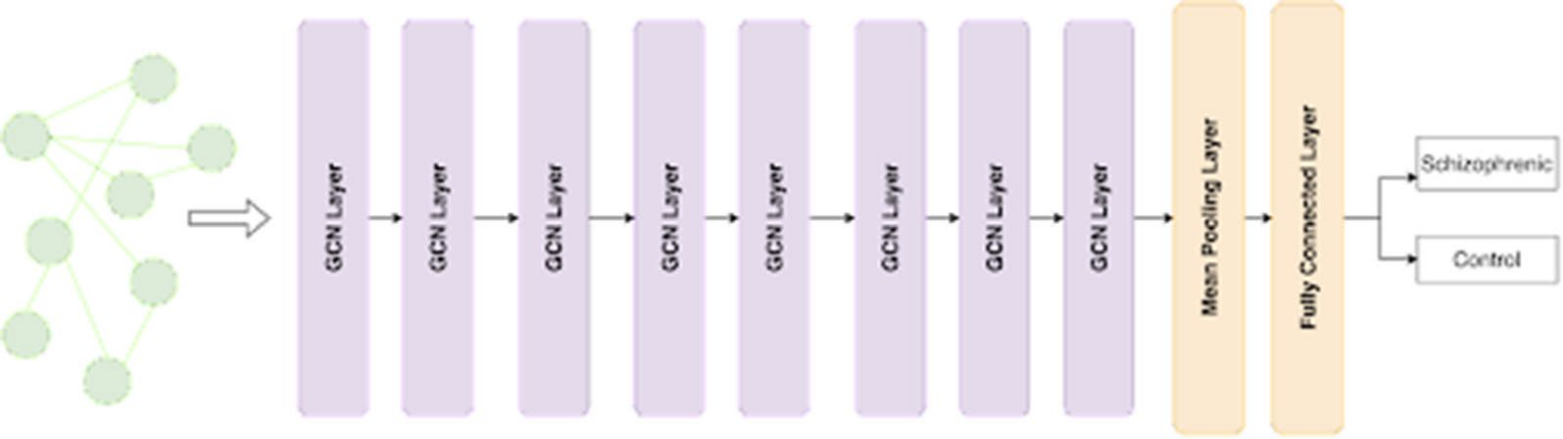
GCN Architecture

The graph convolutional network (GCN) performs semi-supervised learning on graph-structured data that is based on a modified version of the convolutional neural network where the modification allows the model to train on graphs instead of images [30]. The architecture for the same has been displayed in Fig. 8.

#### Deep graph convolutional neural network

**Fig. 9:**
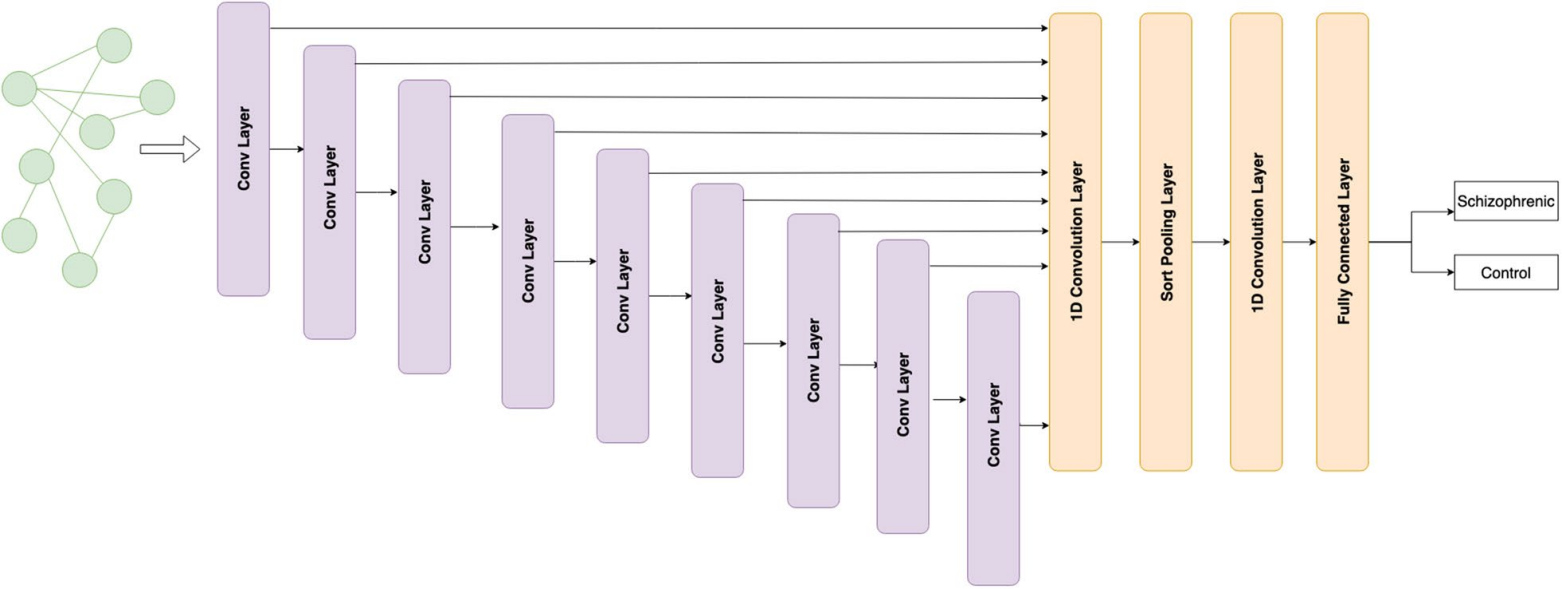
Deep GCN architecture

The DGCNN uses a similar convolutional layer as specified in GCN but with a change in how messages are propagated through layers. DGCNN uses a unique SortPooling technique [15] that sorts nodes in a consistent order as part of the convolution operation, mapping isomorphic graphs to the same output label, which is useful when training graphs with structural differences. The architecture for the same has been displayed in Fig. 9. 10-fold cross-validation was used to evaluate both models.

### Biomarker detection

This paper proposes the following two novel methods to identify potential biomarkers for schizophrenia, the results of which have been displayed in “Salient brain regions” section.

#### Method 1: ROI, local feature pair (RLF) feature selection

We use every pair of (ROI, local feature) as an independent feature resulting in $164 \times 26 = 4264$ features for each subject. Feature selection techniques, namely SHAP and uni-variate feature selection, are used to identify the top 100 RLF pairs that best explain the data, and the most frequently recurring ROIs in these RLF pairs are deemed important.

#### Method 2: Spectral clustering and co-occurrence detection (SpeCo)

With SpeCo, spectral clustering is utilized to segregate the ROIs into clusters. Then, a co-occurrence matrix is created for schizophrenic and control subjects respectively. These matrices, having dimensions of $164\times 164$, record the co-occurrence of all pairs of ROIs in the same cluster for every schizophrenic and control subject respectively. Then, the values are normalized by taking a statistical majority i.e., if the co-occurrence of a certain pair in a cluster exceeds a predefined threshold, then the pair is marked as a co-occurring pair. This method has been run with different number of clusters in the range (2, 5) and different thresholds for the statistical majority in the range (90–95%). The pairs that co-occur for either schizophrenic or control subjects but do not co-occur for both are considered informative for the distinction between a schizophrenic and a non-schizophrenic subject.

## Results

### Classifier performance

We have compared different machine learning classifiers and used GridSearchCV to obtain the best parameters for each ML model. The highest sensitivity obtained is 93.3% (from Decision Tree) and the highest specificity obtained is 96% (from SVM). High sensitivity of the Decision Tree shows that it is desirable in detecting subtle changes in the input features which is crucial for accurate decision-making in medical diagnosis like schizophrenia detection. To address our problem statement, high specificity of the SVM is important to ensure that healthy individuals are not misclassified as having a disease since false positives can lead to unnecessary medical procedures.

In GNN models, the DGCNN performs better than the GCN counterpart, although the difference is not statistically significant. The highest sensitivity obtained for both the GCN and DGCNN is 96% and the highest specificity achieved is 94% (from DGCNN). Owing to its high specificity, the DGCNN demonstrates a strong capability to correctly classify healthy or non-schizophrenic patients as negative, without producing false positive results. Average metrics pertaining to all models can be seen in Table 2.

**Table 2:** Summary of performance of machine learning and GNN models

Model	Accuracy	Specificity	Sensitivity	Precision	F1-score
Logistic regression	62.2 ± 4.2	73.6 ± 10.8	53.2 ± 10.1	67.1 ± 12.5	58.0 ± 5.4
Support vector machine	75.5 ± 4.9	86.2 ± 7.4	65.9 ± 8.9	83.1 ± 9.6	72.8 ± 6.1
K nearest neighbors	67.6 ± 5.6	63.3 ± 8.7	72.5 ± 9.8	66.3 ± 10.4	68.6 ± 7.7
AdaBoost	73.1 ± 7.3	66.3 ± 8.5	80.2 ± 11.4	70.2 ± 10.0	74.3 ± 8.8
Decision tree	78.2 ± 6.7	72.3 ± 8.3	83.3 ± 9.2	75.1 ± 9.3	78.8 ± 8.6
GCN	77.0 ± 2.3	71.4 ± 6.2	82.8 ± 6.2	74.3 ± 3.6	78.1 ± 2.3
DGCNN	80.2 ± 3.3	76.4 ± 8.2	84.2 ± 5.9	77.0 ± 7.7	80.2 ± 4.6

### Salient brain regions

#### RLF pairwise feature selection

RLF pairwise feature selection has yielded the following prominent regions: Supramarginal Gyrus (anterior division), Inferior Temporal Gyrus (posterior, temporooccipital, anterior division), Superior Temporal Gyrus (posterior division, Left), Superior Parietal Lobule (Right), Middle Temporal Gyrus (temporooccipital part, Right).

- The supramarginal gyrus is involved in several cognitive functions and alterations in this region could be relevant to language-related disorders, working memory deficits, or phonological processing impairments [31].
- The inferior temporal gyrus (posterior, temporooccipital) plays a crucial role in visual object recognition and perception, and its significance as a biomarker may relate to visual processing disorders, object recognition deficits, or semantic memory impairments [32].
- The superior temporal gyrus (posterior division, Left) is involved in auditory processing, language comprehension, and speech production [33], and changes in this region could be indicative of auditory processing disorders, speech perception difficulties, or language comprehension deficits.
- The superior parietal lobule (Right) is associated with spatial processing, attention, and sensorimotor integration, and alterations in this region could be relevant to attention deficits, spatial cognition impairments, or motor planning difficulties [34].
- The middle temporal gyrus (temporooccipital part, Right) is involved in various cognitive processes and its significance as a biomarker may relate to semantic processing disorders, language comprehension impairments, or difficulties in integrating visual and linguistic information [35].

#### SpeCo detection

SpeCo (Spectral clustering and co-occurrence) detection has yielded the following prominent pairs of regions: Frontoparietal Right (Posterior Parietal Cortex) and Angular Gyrus Right, Central Opercular Cortex Right and Planum Temporale Left, and Dorsal Attention Left (IPS) and Superior Parietal Lobule Left.

- The posterior parietal cortex and the angular gyrus are involved in various cognitive functions. The significance of the posterior parietal cortex as a biomarker may be related to attentional processes, spatial awareness, or executive functions [36], and that of the angular gyrus right could relate to language-related disorders, spatial cognition deficits, or multi-sensory integration impairments [37].
- The central opercular cortex is responsible for controlling voluntary motor movement and abnormalities or alterations in this region could be indicative of motor-related disorders or impairments [38]. The planum temporale is primarily associated with auditory processing. As a biomarker, changes in the planum temporale may provide insights into language processing abnormalities or auditory-related conditions [39].
- The dorsal attention network, including the intraparietal sulcus (IPS) and superior parietal lobule, is involved in spatial attention. Alterations in the dorsal attention network could be relevant to spatial neglect or attention-related impairments [40].

**Fig. 10:**
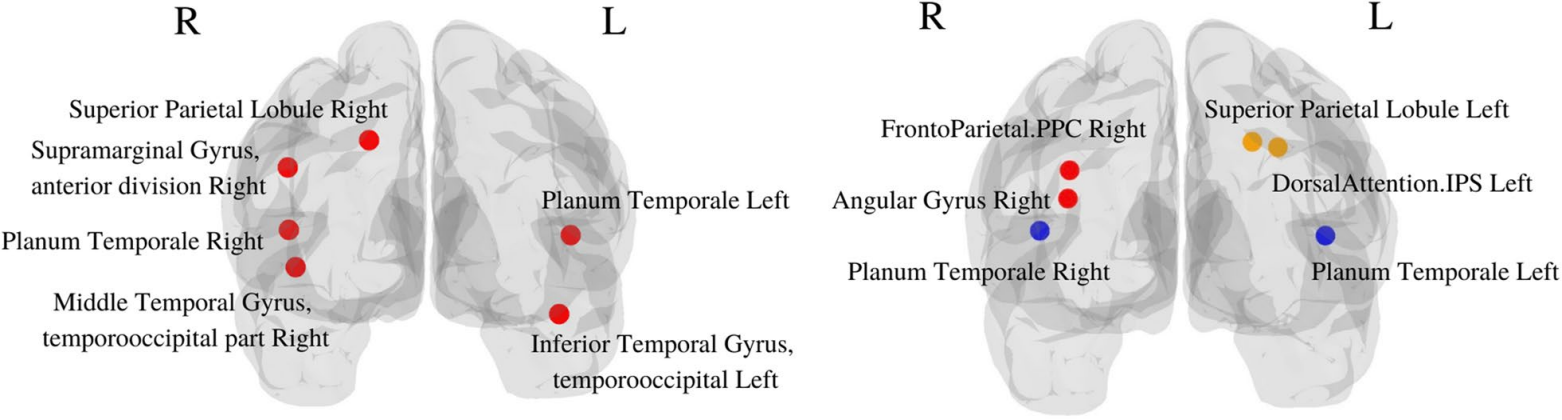
ROIs from RLF feature selection (left) and SpeCo (right)

The important regions from both methods have been displayed in Fig. 10. A detailed list of the biomarkers detected by the two methods can be found in Additional file 1: Sect. S4.

All the code needed to reproduce the results can be found in the supplementary material.

### Interpretability of models

Owing to the sensitive nature of the application, it is important to make these models more understandable by finding the features primarily used for decision-making by these models.

#### SHAP values

**Fig. 11:**
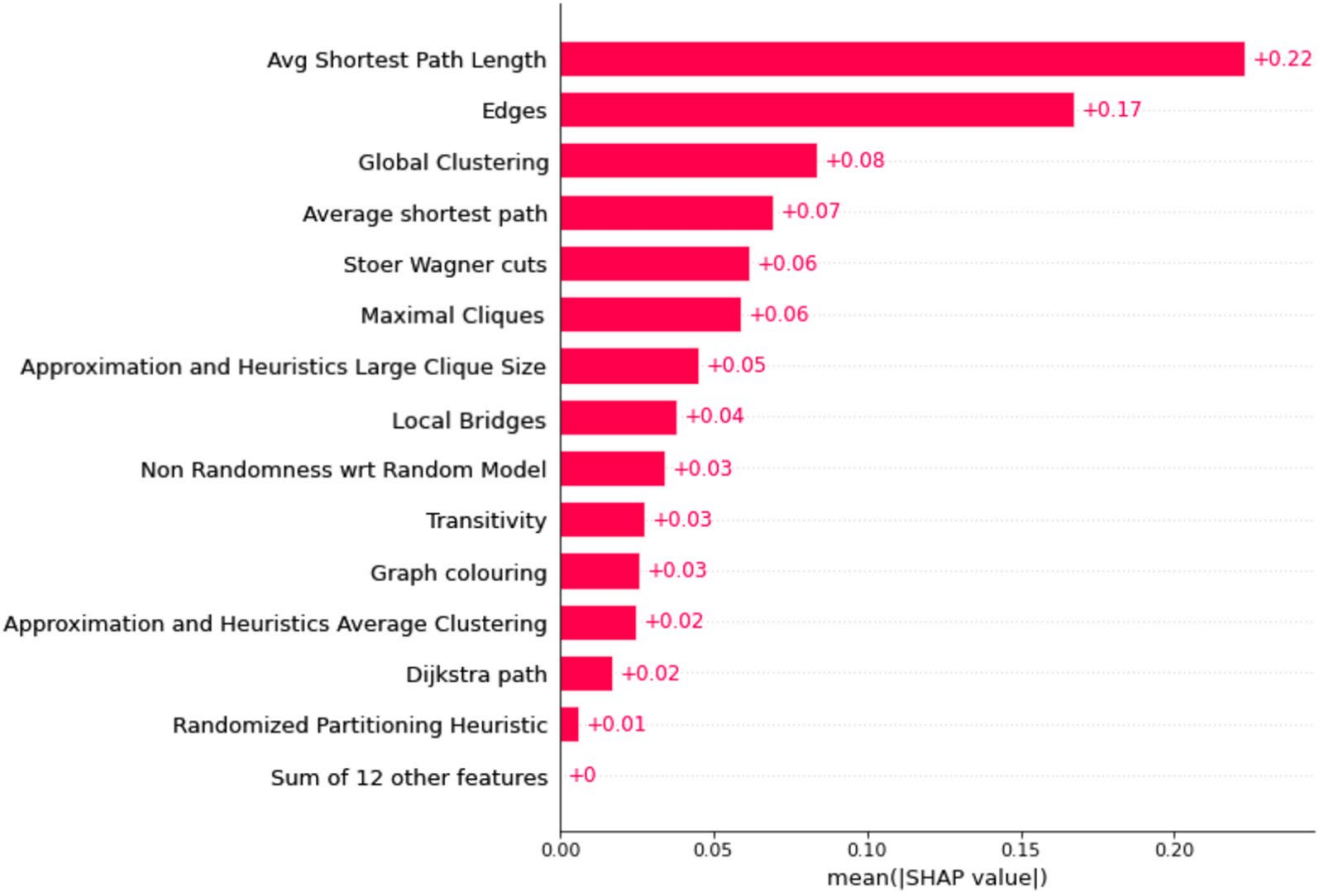
SHAP features

On using SHAP values on tree-based ML methods, the features found to be important along with their percentage contributions are average shortest path length (22%), number of edges (17%), global clustering (8%), average shortest path length (7%), Stoer Wagner cuts (6%), and maximal cliques (6%). The same findings can be visualized in Fig. 11. The average shortest path length indicates the efficiency of information flow, while the number of edges reflects the complexity and interconnectedness of the model [41]. Global clustering reveals patterns of grouped variables, while Stoer Wagner cuts identify subsets with weak connections. Maximal cliques highlight cohesive groups of highly interconnected features [41]. Analyzing these metrics enhances our understanding by uncovering relationships, identifying important features, and improving the interpretability of complex models.

#### GNNExplainer

Similarly, the feature and edge masks of GNNExplainer [42] have been used on the GNN models, resulting in page rank centrality, effective size, greedy coloring, local reaching centrality, and number of cliques as important features. Page Rank centrality identifies influential nodes, while effective size quantifies information spreading ability. Greedy coloring helps identify clusters, and local reaching centrality reveals bridge nodes. The number of cliques indicates structural complexity. By leveraging these features, GNN interpretability improves, enabling a deeper understanding of network structure, influential nodes, information flow, and underlying relationships within the brain graph.

## Discussion

### Optimal binarizing threshold to represent functional networks

Further, to determine which threshold for binarizing the graphs retains the most amount of information relevant to the purpose of classification, this study has also placed emphasis on classification of subjects by considering 9 different binarizing thresholds and comparison amongst the same. The starting value for these thresholds has been taken as 0.0 as it retains only those edges of the graph that have a positive functional correlation, thus eliminating the indefinite nature of biological interpretation for negative correlations [25, 26]. The threshold beyond which this study has not compared the classification prowess of models is 0.3, as the thresholds beyond this value show an increase in isolates in the brain graphs, leading to the inability to calculate the graph measures needed for classification which requires the graphs to have no isolates. A future study based on graph measures that do not have such prerequisites could continue the exploration of these thresholds by considering a higher upper limit on the range of values compared.

### Comparison of ML models and GNN

Drawing a comparison between the graph neural networks and ML models, while each ML model, except Decision Tree, shows a trade-off between specificity and sensitivity, GNNs are able to strike a balance between the two. Moreover, the standard deviations displayed by GNNs, especially in terms of sensitivity and precision, are lower than the ones displayed by the ML models. One possible reason could be that the complex nature and consequently the higher expressive power of the GNN models allow for a more balanced way of classifying the data. Another reason could be the difference in the information captured by the local features combined with the graphs used by the GNNs and the global features used by the ML models. Currently, the DGCNN model slightly outperforms the GCN model, possibly owing to the deeper architecture and unique sort-pooling technique which is better able to capture the topological intricacies of the data. While both ML and GNN models currently perform comparatively, past literature has shown the need for a larger amount of data to adequately train graph neural network models [43]. Hence, access to a larger amount of fMRI data has the potential to improve GNN performance.

### Biomarkers for schizophrenia

In our effort to detect potential biomarkers, we have found that some brain regions found by us are consistent with current literature while others are not as commonly found. Disconnectivity in the Frontopareital region has been reported in studies [44–46] which has also been found by us as an important region. Temporal gyrus (superior, inferior) has also been widely cited as an important biomarker for schizophrenia [32, 47–50] and in our study we found Inferior Temporal Gyrus and Superior Temporal Gyrus to be important contributing factors for schizophrenia detection. Planum Temporale was also found to be an important region in our study and several studies have reported the same [49, 51, 52]. Since our methods have detected several biomarkers that have been previously proven to help in schizophrenia detection, it might be worthwhile conducting further investigation into the other regions detected as biomarkers by our methods that have not been emphasized so far by previous research.

### Supplementary Information

**Additional file 1.** Supplementary Material.

## Data Availability

The fMRI data has been acquired from the publicly available UCLA Consortium for Neuropsychiatric Phenomics LA5c study [22]. The dataset can be downloaded from OpenNeuro repository. More information about the dataset acquisition can be found here. All the preprocessed data along with the code has been made available online to reproduce the results in this paper.

## Code availability

All the preprocessed data along with the code has been made available online to reproduce the results in this paper.
