# Supplementary material for: Graph neural network and machine learning analysis of functional neuroimaging for understanding schizophrenia
Source: BMC Neurosci. 2024 Jan 2;25:2. doi: 10.1186/s12868-023-00841-0 (PMC10759601; doi:10.1186/s12868-023-00841-0)
Supplement: Supplementary file 1 — Additional file 1. Supplementary Material. [file 12868_2023_841_MOESM1_ESM.pdf]

Supplementary material for Graph Neural Network and Machine Learning Analysis of Functional Neuroimaging for Understanding Schizophrenia

Smruthi Gowtham, Gayathri Sunil, Anurita Bose, Samhitha Harish, Gowri Srinivasa

PES Center for Pattern Recognition,
Department of Computer Science and Engineering
PES University
Bengaluru, India

Contents

1 ROI list with coordinates **2**

2 Details about data acquisition **7**

3 Graph measures formulae **8**

3.1 Global Binary 8

3.2 Global Weighted 20

3.3 Local binary 22

3.4 Local weighted 29

4 ML model metrics for different binarizing thresholds **32**

5 Ablation Studies **33**

6 Full list of biomarkers **34**

6.1 Method 1: RLF Feature Selection 34

6.2 Method 2: SpeCo 35

7 Machine Learning Models Hyperparameters **36**

8 GNN with Node2Vec **36**

9 Code **36**

1. ROI list with coordinates

Table 1: ROIs with coordinates

ROI	Region	X	Y	Z
0	DefaultMode.MPFC (1,55,-3)	1	55	-3
1	DefaultMode.LP (L) (-39,-77,33)	-39	-77	33
2	DefaultMode.LP (R) (47,-67,29)	47	-67	29
3	DefaultMode.PCC (1,-61,38)	1	-61	38
4	SensoriMotor.Lateral (L) (-55,-12,29)	-55	-12	29
5	SensoriMotor.Lateral (R) (56,-10,29)	56	-10	29
6	SensoriMotor.Superior (0,-31,67)	0	-31	67
7	Visual.Medial (2,-79,12)	2	-79	12
8	Visual.Occipital (0,-93,-4)	0	-93	-4
9	Visual.Lateral (L) (-37,-79,10)	-37	-79	10
10	Visual.Lateral (R) (38,-72,13)	38	-67	13
11	Salience.ACC (0,22,35)	0	22	35
12	Salience.AInsula (L) (-44,13,1)	-44	13	1
13	Salience.AInsula (R) (47,14,0)	47	14	0
14	Salience.RPFC (L) (-32,45,27)	-32	45	27
15	Salience.RPFC (R) (32,46,27)	32	46	27
16	Salience.SMG (L) (-60,-39,31)	-60	-39	31
17	Salience.SMG (R) (62,-35,32)	62	-35	32
18	DorsalAttention.FEF (L) (-27,-9,64)	-27	-9	64
19	DorsalAttention.FEF (R) (30,-6,64)	30	-6	64
20	DorsalAttention.IPS (L) (-39,-43,52)	-39	-43	52
21	DorsalAttention.IPS (R) (39,-42,54)	39	-42	54
22	FrontoParietal.LPFC (L) (-43,33,28)	-43	33	28
23	FrontoParietal.PPC (L) (-46,-58,49)	-46	-58	49
24	FrontoParietal.LPFC (R) (41,38,30)	41	38	30
25	FrontoParietal.PPC (R) (52,-52,45)	52	-52	45
26	Language.IFG (L) (-51,26,2)	-51	26	2
27	Language.IFG (R) (54,28,1)	54	28	1
28	Language.pSTG (L) (-57,-47,15)	-57	-47	15

Table 1: ROIs with coordinates [continued]

ROI	Region	X	Y	Z
29	Language.pSTG (R) (59,-42,13)	59	-42	13
30	Cerebellar.Anterior (0,-63,-30)	0	-63	-30
31	Cerebellar.Posterior (0,-79,-32)	0	-79	-32
32	FP r (Frontal Pole Right)	26	52	8
33	FP l (Frontal Pole Left)	-25	53	8
34	IC r (Insular Cortex Right)	37	3	0
35	IC l (Insular Cortex Left)	-36	1	0
36	SFG r (Superior Frontal Gyrus Right)	15	18	57
37	SFG l (Superior Frontal Gyrus Left)	-14	19	56
38	MidFG r (Middle Frontal Gyrus Right)	39	19	43
39	MidFG l (Middle Frontal Gyrus Left)	-38	18	42
40	IFG tri r (Inferior Frontal Gyrus, pars triangularis Right)	52	28	8
41	IFG tri l (Inferior Frontal Gyrus, pars triangularis Left)	-50	28	9
42	IFG oper r (Inferior Frontal Gyrus, pars opercularis Right)	52	15	16
43	IFG oper l (Inferior Frontal Gyrus, pars opercularis Left)	-51	15	15
44	PreCG r (Precentral Gyrus Right)	35	-11	50
45	PreCG l (Precentral Gyrus Left)	-34	-12	49
46	TP r (Temporal Pole Right)	41	13	-30
47	TP l (Temporal Pole Left)	-40	11	-30
48	aSTG r (Superior Temporal Gyrus, anterior division Right)	58	-1	-10
49	aSTG l (Superior Temporal Gyrus, anterior division Left)	-56	-4	-8
50	pSTG r (Superior Temporal Gyrus, posterior division Right)	61	-24	2
51	pSTG l (Superior Temporal Gyrus, posterior division Left)	-62	-29	4
52	aMTG r (Middle Temporal Gyrus, anterior division Right)	58	-2	-25
53	aMTG l (Middle Temporal Gyrus, anterior division Left)	-57	-4	-22
54	pMTG r (Middle Temporal Gyrus, posterior division Right)	61	-23	-12
55	pMTG l (Middle Temporal Gyrus, posterior division Left)	-61	-27	-11
56	toMTG r (Middle Temporal Gyrus, temporooccipital part Right)	58	-49	2
57	toMTG l (Middle Temporal Gyrus, temporooccipital part Left)	-58	-53	1

Table 1: ROIs with coordinates [continued]

ROI	Region	X	Y	Z
58	aITG r (Inferior Temporal Gyrus, anterior division Right)	46	-2	-41
59	aITG l (Inferior Temporal Gyrus, anterior division Left)	-48	-5	-39
60	pITG r (Inferior Temporal Gyrus, posterior division Right)	53	-23	-28
61	pITG l (Inferior Temporal Gyrus, posterior division Left)	-53	-28	-26
62	toITG r (Inferior Temporal Gyrus, temporooccipital part Right)	54	-50	-17
63	toITG l (Inferior Temporal Gyrus, temporooccipital part Left)	-52	-53	-17
64	PostCG r (Postcentral Gyrus Right)	38	-26	53
65	PostCG l (Postcentral Gyrus Left)	-38	-28	52
66	SPL r (Superior Parietal Lobule Right)	29	-48	59
67	SPL l (Superior Parietal Lobule Left)	-29	-49	57
68	aSMG r (Supramarginal Gyrus, anterior division Right)	58	-27	38
69	aSMG l (Supramarginal Gyrus, anterior division Left)	-57	-33	37
70	pSMG r (Supramarginal Gyrus, posterior division Right)	55	-40	34
71	pSMG l (Supramarginal Gyrus, posterior division Left)	-55	-46	33
72	AG r (Angular Gyrus Right)	52	-52	32
73	AG l (Angular Gyrus Left)	-50	-56	30
74	sLOC r (Lateral Occipital Cortex, superior division Right)	29	-67	37
75	sLOC l (Lateral Occipital Cortex, superior division Left)	-29	-67	37
76	iLOC r (Lateral Occipital Cortex, inferior division Right)	29	-67	37
77	iLOC l (Lateral Occipital Cortex, inferior division Left)	-29	-67	-37
78	ICC r (Intracalcarine Cortex Right)	12	-74	8
79	ICC l (Intracalcarine Cortex Left)	-10	-75	8
80	MedFC (Frontal Medial Cortex)	0	43	-19
81	SMA r (Juxtapositional Lobule Cortex -formerly Supplementary Motor Cortex- Right)	6	-3	58
82	SMA L(Juxtapositional Lobule Cortex -formerly Supplementary Motor Cortex- Left)	-5	-3	56
83	SubCalC (Subcallosal Cortex)	0	21	-15
84	PaCiG r (Paracingulate Gyrus Right)	7	37	23
85	PaCiG l (Paracingulate Gyrus Left)	-6	37	21
86	AC (Cingulate Gyrus, anterior division)	1	18	24
87	PC (Cingulate Gyrus, posterior division)	1	-37	30

Table 1: ROIs with coordinates [continued]

ROI	Region	X	Y	Z
88	Precuneous (Precuneous Cortex)	1	-59	38
89	Cuneal r (Cuneal Cortex Right)	18	48	14
90	Cuneal l (Cuneal Cortex Left)	-17	47	-13
91	FOrb r (Frontal Orbital Cortex Right)	29	23	-16
92	FOrb l (Frontal Orbital Cortex Left)	-30	24	-17
93	aPaHC r (Parahippocampal Gyrus, anterior division Right)	22	-8	-30
94	aPaHC l (Parahippocampal Gyrus, anterior division Left)	-22	-9	-30
95	pPaHC r (Parahippocampal Gyrus, posterior division Right)	23	-31	-17
96	pPaHC l (Parahippocampal Gyrus, posterior division Left)	-22	-32	-17
97	LG r (Lingual Gyrus Right)	14	-63	-5
98	LG l (Lingual Gyrus Left)	-12	-66	-5
99	aTFusC r (Temporal Fusiform Cortex, anterior division Right)	31	-3	-42
100	aTFusC l (Temporal Fusiform Cortex, anterior division Left)	-32	-4	-42
101	pTFusC r (Temporal Fusiform Cortex, posterior division Right)	36	-24	-28
102	pTFusC l (Temporal Fusiform Cortex, posterior division Left)	-36	-30	-25
103	TOFusC r (Temporal Occipital Fusiform Cortex Right)	32	57	20
104	TOFusC l (Temporal Occipital Fusiform Cortex Left)	-32	57	-20
105	OFusG r (Occipital Fusiform Gyrus Right)	-41	-61	-4
106	OFusG l (Occipital Fusiform Gyrus Left)	39	-59	-6
107	FO r (Frontal Operculum Cortex Right)	41	19	5
108	FO l (Frontal Operculum Cortex Left)	-40	18	5
109	CO r (Central Opercular Cortex Right)	49	-6	11
110	CO l (Central Opercular Cortex Left)	-48	-9	12
111	PO r (Parietal Operculum Cortex Right)	49	-28	22
112	PO l (Parietal Operculum Cortex Left)	-48	-32	20
113	PP r (Planum Polare Right)	48	-4	-7
114	PP l (Planum Polare Left)	-47	-6	-7
115	HG r (Heschl's Gyrus Right)	46	-17	7
116	HG l (Heschl's Gyrus Left)	-45	-20	7

Table 1: ROIs with coordinates [continued]

ROI	Region	X	Y	Z
117	PT r (Planum Temporale Right)	55	-25	12
118	PT l (Planum Temporale Left)	-53	-30	11
119	SCC r (Supracalcarine Cortex Right)	8	-74	14
120	SCC l (Supracalcarine Cortex Left)	-8	-73	15
121	OP r (Occipital Pole Right)	19	60	8
122	OP l (Occipital Pole Left)	-18	61	-8
123	Thalamus r	11	-18	7
124	Thalamus l	-10	-19	6
125	Caudate r	13	10	10
126	Caudate l	-13	9	10
127	Putamen r	25	2	0
128	Putamen l	-25	0	0
129	Pallidum r	20	-4	-1
130	Pallidum l	-19	-5	-1
131	Hippocampus r	26	-21	-14
132	Hippocampus l	-25	-23	-14
133	Amygdala r	23	-4	-18
134	Amygdala l	-23	-5	-18
135	Accumbens r	9	12	-7
136	Accumbens l	-9	11	-7
137	Brain-Stem	0	-30	-35
138	Cereb1 l (Cerebellum Crus1 Left)	-36	-66	-30
139	Cereb1 r (Cerebellum Crus1 Right)	38	-67	-30
140	Cereb2 l (Cerebellum Crus2 Left)	-29	-73	-38
141	Cereb2 r (Cerebellum Crus2 Right)	32	-69	-40
142	Cereb3 l (Cerebellum 3 Left)	-9	-37	-19
143	Cereb3 r (Cerebellum 3 Right)	12	-35	-19
144	Cereb45 l (Cerebellum 4 5 Left)	-14	-44	-17
145	Cereb45 r (Cerebellum 4 5 Right)	16	-44	-19
146	Cereb6 l (Cerebellum 6 Left)	-23	-58	-24
147	Cereb6 r (Cerebellum 6 Right)	24	-58	-25

Table 1: ROIs with coordinates [continued]

ROI	Region	X	Y	Z
148	Cereb7 l (Cerebellum 7b Left)	-32	-60	-45
149	Cereb7 r (Cerebellum 7b Right)	33	-63	-48
150	Cereb8 l (Cerebellum 8 Left)	-26	-55	-49
151	Cereb8 r (Cerebellum 8 Right)	25	-56	-49
152	Cereb9 l (Cerebellum 9 Left)	-11	-49	-46
153	Cereb9 r (Cerebellum 9 Right)	9	-49	-46
154	Cereb10 l (Cerebellum 10 Left)	-23	-34	-42
155	Cereb10 r (Cerebellum 10 Right)	26	-34	-41
156	Ver12 (Vermis 1 2)	1	-39	-20
157	Ver3 (Vermis 3)	1	-40	-11
158	Ver45 (Vermis 4 5)	1	-52	-7
159	Ver6 (Vermis 6)	1	-66	-16
160	Ver7 (Vermis 7)	1	-72	-25
161	Ver8 (Vermis 8)	1	-64	-34
162	Ver9 (Vermis 9)	1	-55	-35
163	Ver10 (Vermis 10)	0	-46	-32

The above coordinates have been referred from [1, 2].

2. Details about data acquisition

The original study contains imaging data for a large group of healthy individuals from the community (138 subjects), as well as samples of individuals diagnosed with schizophrenia (58), bipolar disorder (49), and ADHD (45). For our study, we have taken a sample of 122 control subjects and 50 schizophrenic subjects. We have dropped subjects that had any missing metadata or missing anatomical or functional files.

The ages of participants in the study ranged from 21-50 and belonged to either White, Not of Hispanic or Latino Origin" or "Hispanic or Latino, of Any Race" ethnicity. Participants were screened for neurological disease, history of head injury with loss of consciousness or cognitive sequelae, use of psychoactive medications, substance dependence within the past 6 months, history of major mental illness or ADHD, and current mood or anxiety disorder. Self-reported history of psychopathology was verified with the SCID-IV (First, Spitzer, Gibbon, & Williams, 1995). Urinalysis was used to screen

for drugs of abuse (cannabis, amphetamine, opioids, cocaine, benzodiazepines) on the day of testing and excluded if results were positive. Participants were recruited from the parent study to participate in the fMRI portion if they successfully completed all previous testing sessions, and did not meet the following additional exclusion criteria: history of significant medical illness, contraindications for MRI (including pregnancy), any mood-altering medication on scan day (based on self-report), vision that was insufficient to see task stimuli, and left-handedness.

After receiving a thorough explanation, all participants gave written informed consent according to the procedures approved by the University of California Los Angeles Institutional Review Board.

The fMRI data was recorded on a 3T Siemens Trio scanner. fMRI data was collected using a T2 weighted echoplanar imaging sequence with slice thickness = 4mm, 34 slices, TR = 2s, TE = 30ms and flip angle = 90°. The resting state fMRI scan was recorded for a duration of 304s. The original study contains task aware and resting-state fMRI data but we have only used resting-state fMRI in our study.

3. Graph measures formulae

A comprehensive guide to the formulae and algorithms for the features generated has been detailed below. All measures have been calculated by us using the NetworkX package. All mathematical formulae and code segments have been referred to from [3].

3.1. Global Binary

1. Degree Assortativity:

$$r(x, y) = \sum_{ij} P_i P_j (e_{ij} - a_i b_j)/\sigma_a \sigma_b \tag{1}$$

where,

r is the value for degree assortativity

P is a numerical property assigned to each node

$sigma$ is the standard deviation of P_i

2. Asteroidal Triple:

$$M[u][v] == 0, v \in (u \bigcup N(u)) \tag{2}$$

where,

N(u) is the number of edges from the nodes u

3. Bridges:

A bridge is an edge whose removal increases the number of connected graph components

4. Graph Coloring:

$$P(G,t) = t(t-1)^2(t-2) \tag{3}$$

where,

t are the nodes

5. Average Degree:

$$d_n = \frac{m}{n} \tag{4}$$

where,

m is the number of edges

n is the number of nodes

6. Global Clustering:

$$C = \sum_{i,j,k} A_{ij}A_{jk}A_{ki} / \sum_{i} k_i(k_i - 1) \tag{5}$$

where,

i, j, k are the neighboring clusters

7. Chordal:

$$\sum_{K \in k} |K| = O(|V| + |E|) \tag{6}$$

where,

V are the vertices

E are the edges

8. Transitivity:

$$T = \frac{3 * n_t}{n} \tag{7}$$

where,

n_t is the number of triangles in network

n is the number of connected triples of nodes in the network

9. Approximation and Heuristics Node Connectivity:

```
if nbunch is None:
    nbunch = G
else:
    nbunch = set(nbunch)

directed = G.is_directed()
if directed:
    iter_func = itertools.permutations
else:
    iter_func = itertools.combinations

all_pairs = {n: {} for n in nbunch}

for u, v in iter_func(nbunch, 2):
    k = local_node_connectivity(G, u, v, cutoff=cutoff)
    all_pairs[u][v] = k
    if not directed:
        all_pairs[v][u] = k

return all_pairs
```

10. Approximation and Heuristics Max Independent Sets:

```
def clique_heuristic(G, U, size, best_size):
    if not U:
        return max(best_size, size)
    u = max(U, key=degrees)
    U.remove(u)
    N_prime = {v for v in G[u] if degrees[v] >= best_size}
    return _clique_heuristic(G, U & N_prime, size + 1, best_size)

best_size = 0
nodes = (u for u in G if degrees[u] >= best_size)
for u in nodes:
    neighbors = {v for v in G[u] if degrees[v] >= best_size}
    best_size = _clique_heuristic(G, neighbors, 1, best_size)
return best_size
```

11. Approximation and Heuristics Max Cliques:

```
if G is None:
    raise ValueError("Expected NetworkX graph!")
    cgraph = nx.complement(G)
    iset, _ = clique_removal(cgraph)
return iset
```

12. Approximation and Heuristics Clique Removal:

```
graph = G.copy()
c_i, i_i = ramsey.ramsey_R2(graph)
cliques = [c_i]
isets = [i_i]
while graph:
    graph.remove_nodes_from(c_i)
    c_i, i_i = ramsey.ramsey_R2(graph)
    if c_i:
        cliques.append(c_i)
    if i_i:
        isets.append(i_i)
```

```
maxiset = max(isets, key=len)
return maxiset, cliques
```

13. Approximation and Heuristics Large Clique Size:

```
degrees = G.degree
def _clique_heuristic(G, U, size, best_size):
    if not U:
        return max(best_size, size)
    u = max(U, key=degrees)
    U.remove(u)
    N_prime = {v for v in G[u] if degrees[v] >= best_size}
    return _clique_heuristic(G, U & N_prime, size + 1, best_size)

best_size = 0
nodes = (u for u in G if degrees[u] >= best_size)
for u in nodes:
    neighbors = {v for v in G[u] if degrees[v] >= best_size}
    best_size = _clique_heuristic(G, neighbors, 1, best_size)
return best_size
```

14. Approximation and Heuristics Average Clustering:

```
n = len(G)
triangles = 0
nodes = list(G)
for i in [int(seed.random() * n) for i in range(trials)]:
    nbrs = list(G[nodes[i]])
    if len(nbrs) < 2:
        continue
    u, v = seed.sample(nbrs, 2)
    if u in G[v]:
        triangles += 1
return triangles / trials
```

15. Approximation and Heuristics Diameter:

```
 if not G:
    raise nx.NetworkXError("Expected non-empty NetworkX graph!")
```

```
if G.number_of_nodes() == 1:
    return 0
if G.is_directed():
    return _two_sweep_directed(G, seed)
return _two_sweep_undirected(G, seed)
```

16. Approximation and Heuristics Min Edge Dominating Set:

```
def min_edge_dominating_set(G):
  if not G:
      raise ValueError("Expected non-empty NetworkX graph!")
  return maximal_matching(G)
```

17. Approximation and Heuristics Min Wt Dominating Set:

```
def min_weighted_dominating_set(G, weight=None):
if len(G) == 0:
    return set()
dom_set = set()
def cost(node_and_neighborhood):

    v, neighborhood = node_and_neighborhood
    return G.nodes[v].get(weight, 1) / len(neighborhood - dom_set)
    vertices = set(G)
neighborhoods = {v: {v} | set(G[v]) for v in G}
while vertices:
    dom_node, min_set = min(neighborhoods.items(), key=_cost)
    dom_set.add(dom_node)
    del neighborhoods[dom_node]
    vertices -= min_set
return dom_set
```

18. Approximation and Heuristics Maximal Matching:

The algorithm computes an approximate solution for the minimum maximal cardinality matching problem. The solution is no more than 2 * OPT in size. Runtime is $O(|E|)$.

19. Approximations and Heuristics Ramsey:

```
if not G:
```

```
        return set(), set()
    node = arbitrary_element(G)
    nbrs = (nbr for nbr in nx.all_neighbors(G, node) if nbr != node)
    nnbrs = nx.non_neighbors(G, node)
    c_1, i_1 = ramsey_R2(G.subgraph(nbrs).copy())
    c_2, i_2 = ramsey_R2(G.subgraph(nnbrs).copy())

    c_1.add(node)
    i_2.add(node)
    return max(c_1, c_2, key=len), max(i_1, i_2, key=len)
```

20. Approximation and Heuristics TSP:

```
    dist = {}
    path = {}
    for n, (d, p) in nx.all_pairs_dijkstra(G, weight=weight):
        dist[n] = d
        path[n] = p

    if G.is_directed():
        if not nx.is_strongly_connected(G):
            raise nx.NetworkXError("G is not strongly connected")
        GG = nx.DiGraph()
    else:
        GG = nx.Graph()
    for u in nodes:
        for v in nodes:
            if u == v:
                continue
            GG.add_edge(u, v, weight=dist[u][v])
    best_GG = method(GG, weight)

    if not cycle:
        (u, v) = max(pairwise(best_GG), key=lambda x: dist[x[0]][x[1]])
        pos = best_GG.index(u) + 1
        while best_GG[pos] != v:
```

```
        pos = best_GG[pos:].index(u) + 1
    best_GG = best_GG[pos:-1] + best_GG[:pos]

best_path = []
for u, v in pairwise(best_GG):
    best_path.extend(path[u][v][:-1])
best_path.append(v)
return best_path
```

21. Approximation and Heuristics treewidth Min Degree:

```
def treewidth_min_degree(G):
deg_heuristic = MinDegreeHeuristic(G)
return treewidth_decomp(G, lambda graph: deg_heuristic.best_node(graph))
```

22. Approximation and Heuristics treewidth Min Fill In:

```
def treewidth_min_fill_in(G):
"""Returns a treewidth decomposition using the Minimum Fill-in heuristic.
return treewidth_decomp(G, min_fill_in_heuristic)
```

.

```
return treewidth_decomp(G, min_fill_in_heuristic)
```

23. Approximation and Heuristics treewidth Min Wt Vertex Cover:

```
cost = dict(G.nodes(data=weight, default=1))
cover = set()
for u, v in G.edges():
    if u in cover or v in cover:
        continue
    if cost[u] <= cost[v]:
        cover.add(u)
        cost[v] -= cost[u]
    else:
        cover.add(v)
        cost[u] -= cost[v]
return cover
```

24. Approximation and Heuristics Randomized Partitioning:

```
cut = {node for node in G.nodes() if seed.random() < p}
cut_size = nx.algorithms.cut_size(G, cut, weight=weight)
partition = (cut, G.nodes - cut)
return cut_size, partition
```

25. Approximation and Heuristics One Exchange:

```
f initial_cut is None:
    initial_cut = set()
cut = set(initial_cut)
current_cut_size = nx.algorithms.cut_size(G, cut, weight=weight)
while True:
    nodes = list(G.nodes())
    seed.shuffle(nodes)
    best_node_to_swap = max(
        nodes,
        key=lambda v: nx.algorithms.cut_size(
            G, _swap_node_partition(cut, v), weight=weight
        ),
        default=None,
    )
    potential_cut = _swap_node_partition(cut, best_node_to_swap)
    pot_cutsize = nx.algorithms.cut_size(G, potential_cut, weight=weight)

    if pot_cutsize > current_cut_size:
        cut = potential_cut
        current_cut_size = pot_cutsize
    else:
        break

partition = (cut, G.nodes - cut)
return current_cut_size, partition
```

26. Non-Randomness with respect to Random Model:

```
if not nx.is_connected(G):
```

```
        raise nx.NetworkXException("Non connected graph.")
    if len(list(nx.selfloop_edges(G))) > 0:
        raise nx.NetworkXError("Graph must not contain self-loops")

    if k is None:
        k = len(tuple(nx.community.label_propagation_communities(G)))
    eigenvalues = np.linalg.eigvals(nx.to_numpy_array(G, weight=weight))
    nr = np.real(np.sum(eigenvalues[:k]))

    n = G.number_of_nodes()
    m = G.number_of_edges()
    p = (2 * k * m) / (n * (n - k))
    nr_rd = (nr - ((n - 2 * k) * p + k)) / math.sqrt(2 * k * p * (1 - p))
    return nr, nr_rd
```

27. Number of Connected Components:

```
    seen = set()
    for v in G:
        if v not in seen:
            c = _plain_bfs(G, v)
            seen.update(c)
            yield c
```

28. Diameter:

```
    if usebounds is True and e is None and not G.is_directed():
        return _extrema_bounding(G, compute="diameter")
    if e is None:
        e = eccentricity(G)
    return max(e.values())
```

29. Number of Isolates:

```
        if k < 1:
        raise ValueError(f"k must be positive, not {k}")
    if G.number_of_nodes() < k + 1:
        return False
    elif any(d < k for n, d in G.degree()):
```

```
        return False
    else:
        if k == 1:
            return nx.is_connected(G)
        elif k == 2:
            return not nx.has_bridges(G)
        else:
            return nx.edge_connectivity(G, cutoff=k) >= k
```

30. Number of edges:

```
    if k < 1:
        raise ValueError(f"k must be positive, not {k}")d
    if G.number_of_nodes() < k + 1:
        return False
    elif any(d < k for n, d in G.degree()):
        return False
    else:
        if k == 1:
            return nx.is_connected(G)
        elif k == 2:
            return not nx.has_bridges(G)
        else:
            return nx.edge_connectivity(G, cutoff=k) >= k
```

31. Average shortest path length:

$$a = \sum_{s,t \in V} d(s,t)/n(n-1) \tag{8}$$

where,

V is the set of nodes in G

$d(s,t)$ is the shortest path from s to t

n is the number of nodes in G

32. Average Neighbouring Degree:

```
    andeg = nx.average_neighbor_degree(self.G, weight="weight")
    return andeg
```

33. 1 Edge Connected:

```
deg = {}
for i, u in enumerate(self.G.nodes()):
    deg[u] = 0
    for j, v in enumerate(self.G.nodes()):
        if not self.G.has_edge(u,v):
            pass
        else:
            deg[u]+=self.G.edges[u,v]["weight"]
return deg
```

34. 2 Edge Connected:

```
deg = {}
for i, u in enumerate(self.G.nodes()):
    deg[u] = 0
    for j, v in enumerate(self.G.nodes()):
        if not self.G.has_edge(u,v):
            pass
        else:
            deg[u]+=self.G.edges[u,v]["weight"]
return deg
```

35. Graph Coloring:

```
nodes_to_int = {}
int_to_nodes = {}
for idx, node in enumerate(G.nodes):
        nodes_to_int[node] = idx
        int_to_nodes[idx] = node
G = nx.relabel_nodes(G, nodes_to_int, copy=True)
if len(G.nodes) > 0:
        r_ = max(G.degree(node) for node in G.nodes)
else:
        r_ = 0


if r_ >= num_colors:
```

```
    raise nx.NetworkXAlgorithmError(
        f"Graph has maximum degree {r}, needs "
        f"{r + 1} (> {num_colors}) colors for guaranteed coloring."
    )
```

36. 3 Edge Connected

```
    visited = set()
for u in nodes:
    visited.add(u)
    edges = ((u, v) for u, v, d in H.out_edges(u, data="nontree") if d)
    for u, v in edges:
        chain = list(_build_chain(H, u, v, visited))
        yield chain
```

3.2. Global Weighted

1. Weiner index:

$$W(G) = sum_{i=1}^{i} \sum_{j=1}^{n} d_{ij} = 1/2 \sum_{i=1}^{n} \sum_{j=1}^{n} d_{ij} \tag{9}$$

where,

$W(G)$ is the shortest-path distance between each pair of reachable nodes.

2. Conductance:

$$C = \gamma = \sum_{i \in S, j \in \overline{S}} a_{ij}/min(a(S), a(\overline{S})) \tag{10}$$

where,

S is a collection of nodes

3. Average shortest path length:

$$a = \sum_{s,t \in V} d(s,t)/n(n-1) \tag{11}$$

where,

V is the set of nodes in G

$d(s, t)$ is the shortest path from s to t

n is the number of nodes in G

4. Stoer Wagner index:

```
G = nx.Graph(islice(contractions, best_phase))
v = contractions[best_phase][1]
G.add_node(v)
reachable = set(nx.single_source_shortest_path_length(G, v))
partition = (list(reachable), list(nodes - reachable))

return cut_value, partition
```

5. Randomized Partitioning Heuristic:

```
cut = {node for node in G.nodes() if seed.random() < p}
cut_size = nx.algorithms.cut_size(G, cut, weight=weight)
partition = (cut, G.nodes - cut)
return cut_size, partition
```

6. Max Weight Matching:

```
def max_weight_matching(G, maxcardinality=False, weight="weight"):
    gnodes = list(G)
    if not gnodes:
        return set()
maxweight = 0
allint = True
for i, j, d in G.edges(data=True):
    wt = d.get(weight, 1)
    if i != j and wt > maxweight:
        maxweight = wt
    allint = allint and (str(type(wt)).split("'")[1] in ("int","long"))
```

7. Djikstra Path length:

```
if target is None:
    if method == "unweighted":
```

```
            paths = nx.single_source_shortest_path(G, source)
        elif method == "dijkstra":
            paths = nx.single_source_dijkstra_path(G, source, weight=wt)
        else:  # method == 'bellman-ford':
            paths = nx.single_source_bellman_ford_path(G, source, weight=wt)
    else:
        if method == "unweighted":
            paths = nx.bidirectional_shortest_path(G, source, target)
        elif method == "dijkstra":
            _, paths = nx.bidirectional_dijkstra(G, source, target, wt)
        else:
            paths = nx.bellman_ford_path(G, source, target, wt)
return paths
```

3.3. Local binary

1. **Betweenness centrality:**

$$c_b(v) = \sum_{s,t \in v} \sigma(s,t|v)/\sigma(s,t) \tag{12}$$

where,

s,t are collection of nodes

where,

v is the sum of the fraction of all-pairs shortest paths that pass through

s,t are the collection of nodes

2. **Closeness centrality:**

$$C(u) = n - 1/\sum_{v=1}^{n-1} d(v,u) \tag{13}$$

where,

$d(v,u)$ is the shortest distance between nodes

3. **Eigenvector centrality:**

$$Ax = \lambda x \tag{14}$$

where,

A is the adjacency matrix of the graph G with eigenvalue λ

4. **Average neighbor degree:**

$$deg(x) : x \in N(v) \tag{15}$$

where,

$N(i)$ are the neighbors of node i

5. **Harmonic centrality:**

$$C(u) = \sum_{v \neq u} 1/d(v, u) \tag{16}$$

where,

$d(v, u)$ is the shortest-path distance between v and u

6. **Load centrality:**

```
betweenness = {}
for u, v in G.edges():
    betweenness[(u, v)] = 0.0
    betweenness[(v, u)] = 0.0

for source in G:
    ubetween = _edge_betweenness(G, source, cutoff=cutoff)
    for e, ubetweenv in ubetween.items():
        betweenness[e] += ubetweenv
return betweenness
```

7. **Subgraph centrality:**

$$SC(u) = \sum_{j=1}^{N} (v_j^u)^2 e^{\lambda_j} \tag{17}$$

where,

where v_j is an eigenvector of the adjacency matrix A of G corresponding to the eigenvalue $lambda_j$

8. Square clustering:

$$C_4(v) = \sum_{u=1}^{k_v} \sum_{w=u+1}^{k_v} q_v(u,v) / \sum_{u=1}^{k_v} \sum_{w=u+1}^{k_v} [a_v(u,v) + q_v(u,v)] \tag{18}$$

where,

$q_v(u,w)$ are the number of common neighbors of u and w

9. Information centrality:

Compute current-flow closeness centrality for nodes.

```
information_centrality(G, weight=None, dtype=<class 'float'>,

solver='lu')
```

10. Current flow betweenness centrality:

```
current flow betweenness centrality(G, normalized=True, weight=None,
dtype= [class'float'], solver='full')[source]
```

11. Local Reaching Centrality:

```
if paths is None:
    if nx.is_negatively_weighted(G, weight=weight):
        raise nx.NetworkXError("edge weights must be positive")
    total_weight = G.size(weight=weight)
    if total_weight <= 0:
        raise nx.NetworkXError("Size of G must be positive")
    if weight is not None:
        def as_distance(u, v, d):
            return total_weight / d.get(weight, 1)
```

```
            paths = nx.shortest_path(G, source=v, weight=as_distance)
        else:
            paths = nx.shortest_path(G, source=v)
    if weight is None and G.is_directed():
        return (len(paths) - 1) / (len(G) - 1)
    if normalized and weight is not None:
        norm = G.size(weight=weight) / G.size()
    else:
        norm = 1
    avgw = (_average_weight(G, path, weight=weight) for path in paths.values())
    sum_avg_weight = sum(avgw) / norm
    return sum_avg_weight / (len(G) - 1)
```

12. Node Clique Number:

```
if cliques is None:
        if n is not None:
            if n in G:
                return max(len(c) for c in find_cliques(nx.ego_graph(G,n)))
            return {
            n: max(len(c) for c in find_cliques(nx.ego_graph(G,n)))
                for n in nodes
            }
        cliques = list(find_cliques(G))

    if nodes in G:
        return max(len(c) for c in cliques if nodes in c)

    size_for_n = defaultdict(int)
    for c in cliques:
        size_of_c = len(c)
        for n in c:
            if size_for_n[n] < size_of_c:
                size_for_n[n] = size_of_c
    if nodes is None:
        return size_for_n
```

```
    return {n: size_for_n[n] for n in nodes}
```

13. **Number of Cliques:**

```
    if cliques is None:
        cliques = list(find_cliques(G))


    if nodes is None:
        nodes = list(G.nodes())


    if not isinstance(nodes, list):
        v = nodes
        numcliq = len([1 for c in cliques if v in c])
    else:
        numcliq = {}
        for v in nodes:
            numcliq[v] = len([1 for c in cliques if v in c])
    return numcliq
```

14. **Greedy Coloring:**

```
    if interchange:
    if strategy is strategy_independent_set:
        msg = "interchange cannot be used with independent_set"
        raise nx.NetworkXPointlessConcept(msg)
    if strategy is strategy_saturation_largest_first:
        msg = "interchange cannot be used with" "saturation_largest_first"
        raise nx.NetworkXPointlessConcept(msg)
colors = {}
nodes = strategy(G, colors)
if interchange:
    return _greedy_coloring_with_interchange(G, nodes)
for u in nodes:
    neighbour_colors = {colors[v] for v in G[u] if v in colors}
    for color in itertools.count():
        if color not in neighbour_colors:
            break
```

```
        colors[u] = color
return colors
```

15. Page Rank Centrality:

```
    return pagerank_scipy(
        G, alpha, personalization, max_iter, tol, nstart, weight, dangling
    )
```

16. Degree:

```
def degree(G, nbunch=None, weight=None):
    """Returns a degree view of single node or of nbunch of nodes.
    If nbunch is omitted, then return degrees of *all* nodes.
    """
    return G.degree(nbunch, weight)
```

17. Degree Centrality:

```
    if len(G) <= 1:
        return {n: 1 for n in G}

    s = 1.0 / (len(G) - 1.0)
    centrality = {n: d * s for n, d in G.degree()}
    return centrality
```

18. Local Clustering:

Local clustering is sometimes used as a measure of centrality, as well as a way to study the large-scale structure of a network. Here we discuss clustering as a centrality measure.

19. Eccentricity:

```
    for n in G.nbunch_iter(v):
        if sp is None:
            length = nx.single_source_shortest_path_length(G, n)
            L = len(length)
        else:
            try:
                length = sp[n]
                L = len(length)
```

```
        except TypeError as err:
            raise nx.NetworkXError('Format of "sp" is invalid.') from err
    if L != order:
        if G.is_directed():
            msg = (
                "Found infinite path length because the digraph is not"
                " strongly connected"
            )
        else:
            msg = "Found infinite path length"
        raise nx.NetworkXError(msg)

    e[n] = max(length.values())

if v in G:
    return e[v]
else:
    return e
```

20. Second-Order Clustering:

$$c_u = \sum_{v \in N(N(u))} c_{uv} / |N(N(u))| \tag{19}$$

where,

$N(u)$ is the number of edges from the nodes

21. Approx. current flow betweenness centrality:

```
    n = G.number_of_nodes()
ordering = list(reverse_cuthill_mckee_ordering(G))
H = nx.relabel_nodes(G, dict(zip(ordering, range(n))))
L = nx.laplacian_matrix(H, nodelist=range(n), weight=weight).asformat("csc")
L = L.astype(dtype)
C = solvername[solver](L, dtype=dtype)
betweenness = dict.fromkeys(H, 0.0)
nb = (n - 1.0) * (n - 2.0)
```

```
cstar = n * (n - 1) / nb
l = 1
k = l * int(np.ceil((cstar / epsilon) ** 2 * np.log(n)))
if k > kmax:
    msg = f"Number random pairs k>kmax ({k}>{kmax}) "
    raise nx.NetworkXError(msg, "Increase kmax or epsilon")
cstar2k = cstar / (2 * k)
for _ in range(k):
    s, t = pair = seed.sample(range(n), 2)
    b = np.zeros(n, dtype=dtype)
    b[s] = 1
    b[t] = -1
    p = C.solve(b)
    for v in H:
        if v in pair:
            continue
        for nbr in H[v]:
            w = H[v][nbr].get(weight, 1.0)
            betweenness[v] += w * np.abs(p[v] - p[nbr]) * cstar2k
if normalized:
    factor = 1.0
else:
    factor = nb / 2.0
return {ordering[k]: v * factor for k, v in betweenness.items()}
```

3.4. Local weighted

1. Closeness Vitality:

```
n = G.number_of_nodes()
ordering = list(reverse_cuthill_mckee_ordering(G))
H = nx.relabel_nodes(G, dict(zip(ordering, range(n))))
betweenness = dict.fromkeys(H, 0.0)
n = H.number_of_nodes()
L = nx.laplacian_matrix(H, nodelist=range(n), weight=weight).asformat("csc")
L = L.astype(dtype)
```

```python
    C2 = solvername[solver](L, width=1, dtype=dtype)
    for v in H:
        col = C2.get_row(v)
        for w in H:
            betweenness[v] += col[v] - 2 * col[w]
            betweenness[w] += col[v]
    for v in H:
        betweenness[v] = 1 / (betweenness[v])
    return {ordering[k]: v for k, v in betweenness.items()}
```

2. Constraint:

$$c(v) = \sum_{w \in N(v)} l(v, w) \tag{20}$$

where,

$N(v)$ is the subset of neighbours

$l(v, w)$ are local constraints on v and w

3. Effective Size:

$$e(u) = \sum_{w \in N(u)} (1 - \sum_{w \in N(v)} p_{uw} m_{vw}) \tag{21}$$

where,

$N(v)$ is the set of neighbours

m_{vw} is the mutual weight of neighbours

4. Average neighbor degree:

```python
for n, deg in source_degree(nodes, weight=weight):
    if deg == 0:
        avg[n] = 0.0
        continue

    if weight is None:
        avg[n] = (
            sum(t_deg[nbr] for nbr in G_S[n])+sum(t_deg[nbr] for nbr in G_P[n])
```

```
        ) / deg
    else:
        avg[n] = (
            sum(dd.get(weight, 1)*t_deg[nbr] for nbr, dd in G_S[n].items())
            + sum(dd.get(weight, 1)*t_deg[nbr] for nbr, dd in G_P[n].items())
        ) / deg
return avg
```

5. Weighted degree:

```
deg = {}
for i, u in enumerate(self.G.nodes()):
  deg[u] = 0
  for j, v in enumerate(self.G.nodes()):
    if not self.G.has_edge(u,v):
      pass
    else:
      deg[u]+=self.G.edges[u,v]["weight"]
return deg
```

4. ML model metrics for different binarizing thresholds

Table 2: Metrics for various binarizing thresholds

Threshold	Model	Accuracy	Specificity	Sensitivity	Precision	F1-score
0.00	RF	70.5±7.8	80.0±6.6	31.4±13.3	59.2±21.7	40.6±13.0
	AB	70.4± 7.7	84.6±8.2	36.0±12.8	51.8±16.4	41.0±11.7
	XGB	68.6±7.8	88.2±7.9	20.6±12.3	44.3±16.6	26.3±12.9
0.05	RF	71.4±7.4	85.5±9.2	33.9±12.0	57.5±22.3	42.5±9.8
	AB	72.2±7.4	89.4±8.3	33.1±14.5	60.0±22.1	39.2±13.1
	XGB	70.6±6.0	88.6±6.7	28.1±11.6	51.6±18.2	33.7±8.9
0.10	RF	71.7±7.7	80.4±6.9	26.5±14.6	54.1±16.3	33.5±12.9
	AB	70.6±10.9	87.3±8.1	32.6±17.6	49.9±27.8	37.7±19.3
	XGB	68.3±9.5	87.9±10.1	21.2±13.1	44.5±17.6	26.8±12.8
0.15	RF	75.3±6.2	86.0±4.8	24.2±8.9	61.4±39.0	34.2±13.1
	AB	74.5±5.4	85.7±5.0	46.0±9.8	55.4±12.8	49.5±8.5
	XGB	72.3±7.9	89.7±8.6	17.8±8.9	33.6±22.9	15.6±12.2
0.20	RF	73.± 7.6	88.8±7.8	36.7±15.4	58.3±18.0	44.4±14.8
	AB	77.9±7.2	88.0±5.6	48.5±16.2	58.2±10.9	51.1±10.7
	XGB	67.8±8.1	89.3±9.0	15.8±7.0	42.1±23.1	26.8±12.8
0.25	RF	71.9±4.9	90.7±6.6	29.9±10.1	46.1±24.7	36.3±12.1
	AB	75.3±6.5	85.9±6.5	47.0±17.8	55.6±19.3	49.5±15.3
	XGB	75.1±5.2	92.2±6.8	28.1±7.7	66.3±26.1	37.1±9.6
0.30	RF	73.0±6.1	93.9±4.5	12.8±10.5	52.3±25.0	20.8±13.2
	AB	75.9±8.0	93.7±6.6	22.8±12.5	60.8±33.7	32.0±16.1
	XGB	67.3±8.7	89.5±8.4	14.1±9.8	37.7±24.9	19.7±13.4

No	Variant	#layers	LR	Epoch	Accuracy	Precision	Recall	F1 score	Specificity
1	DGCNN	5	0.0005	50	0.68 ± 0.03	0.34 ± 0.07	0.85 ± 0.13	0.48 ± 0.06	0.94 ± 0.05
2	DGCNN	7	0.0005	50	0.7 ± 0.01	0.38 ± 0.08	0.88 ± 0.09	0.52 ± 0.06	0.95 ± 0.05
3	DGCNN	9	0.0005	50	0.71 ± 0.047	0.42 ± 0.07	0.87 ± 0.14	0.56 ± 0.071	0.94 ± 0.07
4	DGCNN	10	0.0005	50	0.76 ± 0.08	0.65± 0.09	0.88 ± 0.09	0.70 ± 1.6	0.95 ± 0.10
5	DGCNN	12	0.0005	50	0.69 ± 0.02	0.38 ± 0.08	0.86 ± 0.09	0.48 ± 0.6	0.89 ± 0.8
6	DGCNN	10	0.0005	80	0.76 ± 0.12	0.65 ± 0.09	0.88 ± 0.09	0.72 ± 1.6	0.95 ± 0.10
7	DGCNN	10	0.0005	100	0.78 ± 0.08	0.68 ± 0.1	0.89 ± 0.09	0.76 ± 1.4	0.95 ± 0.10
8	DGCNN	10	0.005	100	0.78 ± 0.05	0.66 ± 0.18	0.9 ± 0.07	0.75 ± 0.11	0.94 ± 0.06
9	GCN	5	0.0005	100	0.66 ± 0.08	0.33 ± 0.01	0.88 ± 0.08	0.50 ± 0.11	0.93 ± 0.13
10	GCN	7	0.0005	100	0.7 ± 0.03	0.36 ± 0.07	0.91 ± 0.08	0.52 ± 0.08	0.95 ± 0.09
11	GCN	9	0.0005	100	0.74 ± 0.02	0.60 ± 0.04	0.94 ± 0.05	0.64 ± 0.03	0.96 ± 0.08
12	GCN	9	0.005	100	0.76 ± 0.01	0.62 ± 0.04	0.96 ± 0.04	0.63 ± 0.03	0.98 ± 0.02

5. Ablation Studies

A description of each variant and its mapping is given below:

No.	Variant	Number of layers	Layers	Activations
1	DGCNN	5	[20, 32, 16, 4, 2]	["leaky_relu" *4, "softmax"]
2	DGCNN	7	[20, 32, 64, 32, 16, 4, 2]	["leaky_relu" *6, "softmax"]
3	DGCNN	9	[20, 32, 64, 128, 64, 32, 16, 4, 2]	["leaky_relu" *8, "softmax"]
4	DGCNN	10	[20, 32, 64, 128, 128, 64, 32, 16, 4, 2]	["leaky_relu" *9, "softmax"]
5	DGCNN	12	[20, 32, 64, 128, 256, 128, 64, 32, 16, 4, 2]	["leaky_relu" *11, "softmax"]
6	DGCNN	10	[20, 32, 64, 128, 128, 64, 32, 16, 4, 2]	["leaky_relu" *9, "softmax"]
7	DGCNN	10	[20, 32, 64, 128, 128, 64, 32, 16, 4, 2]	["leaky_relu" *9, "softmax"]
8	DGCNN	10	[20, 32, 64, 128, 128, 64, 32, 16, 4, 2]	["leaky_relu" *9, "softmax"]
9	GCN	5	[20, 32, 64, 16, 2]	["leaky_relu" *4, "softmax"]
10	GCN	7	[20, 32, 64, 32, 16, 4, 2]	["leaky_relu" *6, "softmax"]
11	GCN	9	[20, 32, 64, 128, 64, 32, 16, 4, 2]	["leaky_relu" *8, "softmax"]
12	GCN	9	[20, 32, 64, 128, 64, 32, 16, 4, 2]	["leaky_relu" *8, "softmax"]

6. Full list of biomarkers

6.1. Method 1: RLF Feature Selection

The following is the list of potential biomarkers found using the integrated local ROI, feature pairwise analysis

- aSMG r (Supramarginal Gyrus, anterior division Right)
- Inferior Temporal Gyrus, posterior division Right
- Frontal Medial Cortex
- Superior Parietal Lobule Right
- toITG l (Inferior Temporal Gyrus, temporooccipital part Left)
- pSTG l (Superior Temporal Gyrus, posterior division Left)
- pITG l (Inferior Temporal Gyrus, posterior division Left)
- CO l (Central Opercular Cortex Left)
- Cereb2 r (Cerebelum Crus2 Right)
- PreCG r (Precentral Gyrus Right)
- PreCG r (Precentral Gyrus Right)

- Putamen l
- toMTG r (Middle Temporal Gyrus, temporooccipital part Right)
- OFusG l (Occipital Fusiform Gyrus Left)
- FrontoParietal.LPFC (R) (41,38,30)

6.2. Method 2: SpeCo

The following are the lists of pairs of regions of interest (ROIs) obtained as potential biomarkers from the spectral clustering method:

Number of clusters = 2

Regions:

- Cerebellar.Posterior and Cereb2 l (Cerebelum Crus2 Left)
- IC r (Insular Cortex Right) and IC l (Insular Cortex Left)
- PreCG r (Precentral Gyrus Right) and PreCG l (Precentral Gyrus Left)
- CO r (Central Opercular Cortex Right) and CO l (Central Opercular Cortex Left)
- CO r (Central Opercular Cortex Right) and PT l (Planum Temporale Left)
- CO l (Central Opercular Cortex Left) and PT r (Planum Temporale Right)
- PO l (Parietal Operculum Cortex Left) and PT l (Planum Temporale Left)
- PP r (Planum Polare Right) and PP l (Planum Polare Left)
- PT r (Planum Temporale Right) and PT l (Planum Temporale Left)

Number of clusters = 3

Regions:

- SensoriMotor.Lateral (L) and SensoriMotor.Lateral (R)
- Visual.Medial and pSTG l (Superior Temporal Gyrus, posterior division Left)
- DorsalAttention.IPS (L) and SPL l (Superior Parietal Lobule Left)
- FrontoParietal.PPC (R) and AG r (Angular Gyrus Right)
- Cerebellar.Posterior and Cereb1 r (Cerebelum Crus1 Right)
- OFusG r (Occipital Fusiform Gyrus Right) and OFusG l (Occipital Fusiform Gyrus Left)
- PT r (Planum Temporale Right) and PT l (Planum Temporale Left)

7. Machine Learning Models Hyperparameters

- SVM- 'C': 1, 'gamma': 0.0001, 'kernel': 'rbf'
- KNN- 'n_neighbors': 3, 'p': 1
- Decision Tree- 'criterion': 'entropy', 'max_depth': 19
- AdaBoost 'learning_rate': 1.0, 'n_estimators': 93

8. GNN with Node2Vec

We have also explored the option of adding Node2Vec features along with local features as input for the GNN. We used Node2Vec to additionally generate 8 features for each node via the Skip-gram technique. This generates a vector representation for each node in the graph based on a random walk conducted on the neighborhood of the node and can be used in downstream tasks for the purpose of learning patterns.

Taking a closer look at Table 2, we can summarize that Node2Vec features neither significantly enhance nor diminish the classifying capability of GNNs. This implies that the characteristics of the functional connectivity of the brain graph captured by Node2Vec are either not significant to the purposes of this study, or that they are already captured by GNNs and are hence rendered moot. Future studies could verify the latter hypothesis by using models other than GNN for classifying schizophrenic and control subjects based on Node2Vec, as obtaining a similar result, in this case, could indicate that Node2Vec achieves, with less computational resources, a comparable diagnostic performance as a complex and resource-intensive GNN does. This would also speak to the importance of the structural aspect of the brain graph for the purpose of classification.

No.	Model	Accuracy	Specificity	Sensitivity	Precision	F1-score
2	GCN with n2v	76.8±4.0	76.0±4.9	77.5±7.5	76.4±3.7	76.8±4.5
3	DGCNN	80.2±3.3	76.4±8.2	84.2±5.9	77.0±7.7	80.2±4.6

Table 2: Performance of Node2Vec with GNN

9. Code

All the code files and data have been made available here.

References

[1] S. Whitfield-Gabrieli, A. Nieto-Castanon, Conn: a functional connectivity toolbox for correlated and anticorrelated brain networks, Brain connectivity 2 (3) (2012) 125–141.

[2] S. Shojaeilangari, N. Radman, M. E. Taghizadeh, H. Soltanian-Zadeh, rsfmri based evidence for functional connectivity alterations in adults with developmental stuttering, Heliyon 7 (9) (2021) e07855.

[3] A. A. Hagberg, D. A. Schult, P. J. Swart, Exploring network structure, dynamics, and function using networkx, in: G. Varoquaux, T. Vaught, J. Millman (Eds.), Proceedings of the 7th Python in Science Conference, Pasadena, CA USA, 2008, pp. 11 – 15.
